# Physiological responses in free-ranging Asian elephant populations living across human-production landscapes

**DOI:** 10.1038/s41598-025-06243-y

**Published:** 2025-09-04

**Authors:** Sanjeeta Sharma Pokharel, Amir Kumar Chettri, Sunipa Chatterjee, Polani B. Seshagiri, Raman Sukumar

**Affiliations:** 1 Centre for Ecological Sciences, Indian Institute of Science, Bangalore, India; 2 Graduate School of Asian and African Area Studies, Kyoto University, Kyoto, Japan; 3 Present Address: The Hakubi Center for Advanced Research, Kyoto University, Kyoto, Japan; 4 Department of Developmental Biology and Genetics, Indian Institute of Science, Bangalore, India; 5 The Co-existence Project, Kolkata, West Bengal India

**Keywords:** Faecal thyroid hormones, Stress physiology, *Elephas maximus*, Fragmentation, Triiodothyronine, Glucocorticoids, Faecal C/N ratio, Ecology, Physiology

## Abstract

Monitoring the physiology of elephants living in human-production landscapes has become increasingly important for understanding how they cope with various challenges that affect their overall fitness. We assessed physiological stress by measuring faecal glucocorticoid metabolite (fGCM) levels and metabolic states using faecal triiodothyronine (fT3) across three free-ranging Asian elephant populations (one in Central India and two in Northeastern India) whose home ranges encompass varying extents of disturbance in human-production landscapes. We present landscape disturbance metrics to characterize variations in fragmentation and anthropogenic pressures across the study landscapes and use faecal carbon and nitrogen (C/N) ratio as a proxy for dietary quality, with higher C/N values indicating poorer-quality diets. Elephants living in more fragmented habitats in Central India had higher fGCM and lower fT3 levels compared to the Northeastern populations, as well as when compared (only fGCM levels) with a previously-studied Southern Indian elephant population. A positive relationship was observed between faecal C/N ratio and fGCM levels across the populations, except for the Central population. These findings suggest that elephants in highly fragmented landscapes and experiencing significant anthropogenic disturbances have (i) higher adrenal activity to cope with and (ii) reduced metabolic rates to conserve energy in emerging challenging contexts. While elephants may adapt to living in human-modified landscapes to some extent, they may experience high stress levels beyond a threshold of disturbance which can be physiologically costly. This warrants systematic assessments to evaluate how these biological costs impact their fitness, and a re-evaluation of conflict management practices.

**Supplementary Information:**

The online version contains supplementary material available at 10.1038/s41598-025-06243-y.

## Introduction

Decline in habitat quality, driven by human alterations and climate-related events, has adversely affected the survival of a number of animal taxa^1,2^. Asian elephants are among the large mammals facing high risks to their survival due to habitat loss and fragmentation, with agricultural expansion leading to a 64% reduction in potential elephant habitats across Asia since c.1700 CE^3,4^. Studies show that these elephants are adjusting their behaviours, such as foraging, social organization, vocal communication, and ranging patterns, in response to human-altered landscapes^5–8^. Such behavioural adaptability may stem from internal physiological adjustments^9^. It is essential to explore whether elephants also ‘adapt’ physiologically and metabolically to life in human-production landscapes and the subsequent consequences for their overall fitness.

The physiological health of elephants can be assessed by measuring one of the vital components of the stress response, glucocorticoids (GCs), which reflect different states of biological response towards environmental stressors^10–12^. Changes in GC levels are often linked to environmentally-challenging situations, thus making them useful indicators for studying how animals physiologically adjust to altered landscapes and human disturbances^12^. The relative ease of measuring GC metabolites in animals’ faeces (faecal glucocorticoid metabolites or fGCM) to assess the adrenocortical activity non-invasively makes this a practical technique, particularly for free-ranging elephants^13^.

Similarly, thyroid hormones (THs), mainly the metabolically active triiodothyronine (T3 or 3’,3,5-triiodothyronine) and its levels in faeces (faecal triiodothyronine or fT3), have been widely used to monitor metabolic states and ontogenetic functions at various life stages across different mammalian species, including elephants^14 − 18^. The hypothalamic-pituitary-thyroid (HPT) axis plays a key role in THs synthesis and regulation, similar to the hypothalamic-pituitary-adrenal (HPA) axis for GCs^16,17^. THs are critical for regulating metabolic activity and energy expenditure concerning challenging physiological contexts (e.g. resource deprivation, extreme temperatures, pregnancy)^14,15^. Studies on different mammalian species indicate that THs are significantly influenced by contexts associated with energy homeostasis, such as nutritional deficits, starvation, anthropogenic disturbances, temperature, weight gain, lactation and sexual maturity^14,15,18–25^.

In mammals, the activation of the HPA axis is linked to decreased THs signalling, reduced secretion of thyroid-stimulating hormones (TSH) and, consequently, lowered production of THs^14,15^. While there are studies documenting the links between GCs and various stressors in free-ranging elephants^8^the relationships between THs and GCs in elephants are relatively less studied (Table S1). With limited studies on THs in elephants (mainly on captive elephants: Asian^20,26^; African^27^; both species^25,28,29^; free-ranging: Asian^20^African^19^; Table S1), our understanding of how metabolic and adrenocortical activities interact in the natural context or in response to anthropogenic stimuli, particularly in elephants living in human-production landscapes, remains limited.

India’s estimated free-ranging elephant population of 27,000 to 30,000 individuals, is distributed across four major geographical regions – Northeastern, Northwestern, Central and Southern – with varying degrees of habitat fragmentation and elephant-human negative interactions (EHN) leading to property damage, elephant and human mortalities, and large-scale depredation of crops^30^. The elephant populations in West Bengal state, representing both the Northeastern and Central Indian landscapes which are highly fragmented, experience among the highest levels of such conflicts in the country^31,32^.

Our earlier research revealed that crop-foraging elephants residing in a human-production landscape had lower levels of fGCM compared to elephants living in adjoining protected habitats, possibly due to superior nutrition from the crops masking any additional stress experienced by elephants in the former landscape^5^. Since the study was conducted in a single Southern population in India with relatively lower anthropogenic disturbances, the present study aimed to advance these insights to other Indian landscapes representing varying degrees of EHN. Apart from the nature of habitat disturbance (degradation, conversion to monocultures, fragmentation), the type of management responses to crop foraging elephants varies substantially from relatively passive to more active or even aggressive methods. Thus, habitat quality and human responses can be expected to influence stress levels in elephants.

We studied the differences in metabolic activity (specifically fT3; which has not been previously examined in free-ranging Asian elephants in relation to human disturbances) and adrenocortical activities (measured by fGCM) among three free-ranging populations of elephants in India, while keeping other factors such as seasonality, crop foraging, and age classes constant or relatively similar for these populations. We then compared these results with those from a population in the Southern India^5^. We measured faecal nitrogen (N) and carbon (C) contents and used faecal C/N ratio for each sample as a surrogate measure of dietary quality^5^. We also provided landscape disturbance metrics for the four elephant populations to contextually and quantitatively illustrate the extent of habitat fragmentation and anthropogenic disturbances such as human retaliatory responses and conflict management practices, across these landscapes (see Methods section). We predicted that elephants living in more fragmented landscapes, as measured by various landscape disturbance metrics, may be at higher risk of experiencing anthropogenic disturbances or poorer dietary quality (higher faecal C/N ratio) leading to: (i) higher adrenocortical activity (reflected by higher fGCM levels) and (ii) a compromised metabolic state (altered energy homeostasis as indicated by lower fT3).

## Results

**Fig. 1:**
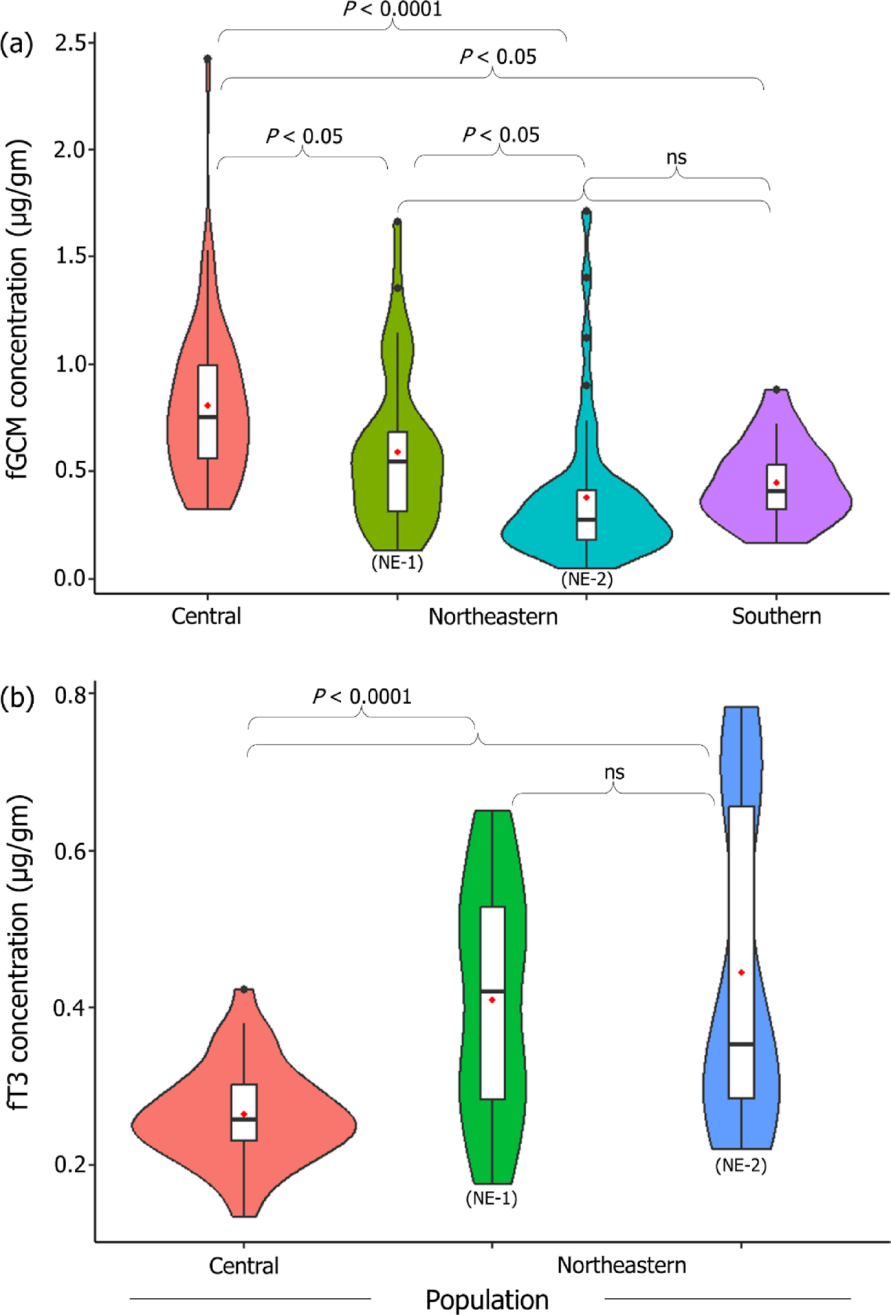
fGCM and fT3 concentrations in free-ranging Asian elephants across populations in human-production landscapes. Violin graphs of grouped concentrations of (a) fGCM (µg/g; *n* = 153) in elephants from the Northeastern (including NE-1 and NE-2), Central and Southern populations, and (b) fT3 (µg/g; *n* = 107) in elephants from the Northeastern and Central populations. The boxplots within the violins indicate the median, with the mean as a red dot, and the upper and lower quartiles. The shaded regions of the violin plots represent data distribution, smoothened using a probability density function. Statistical significance (denoted as *P* for significant and ns for nonsignificant *p*-values) was assessed GLMs (Tables 1 and 2, and S2).

**Fig. 2:**
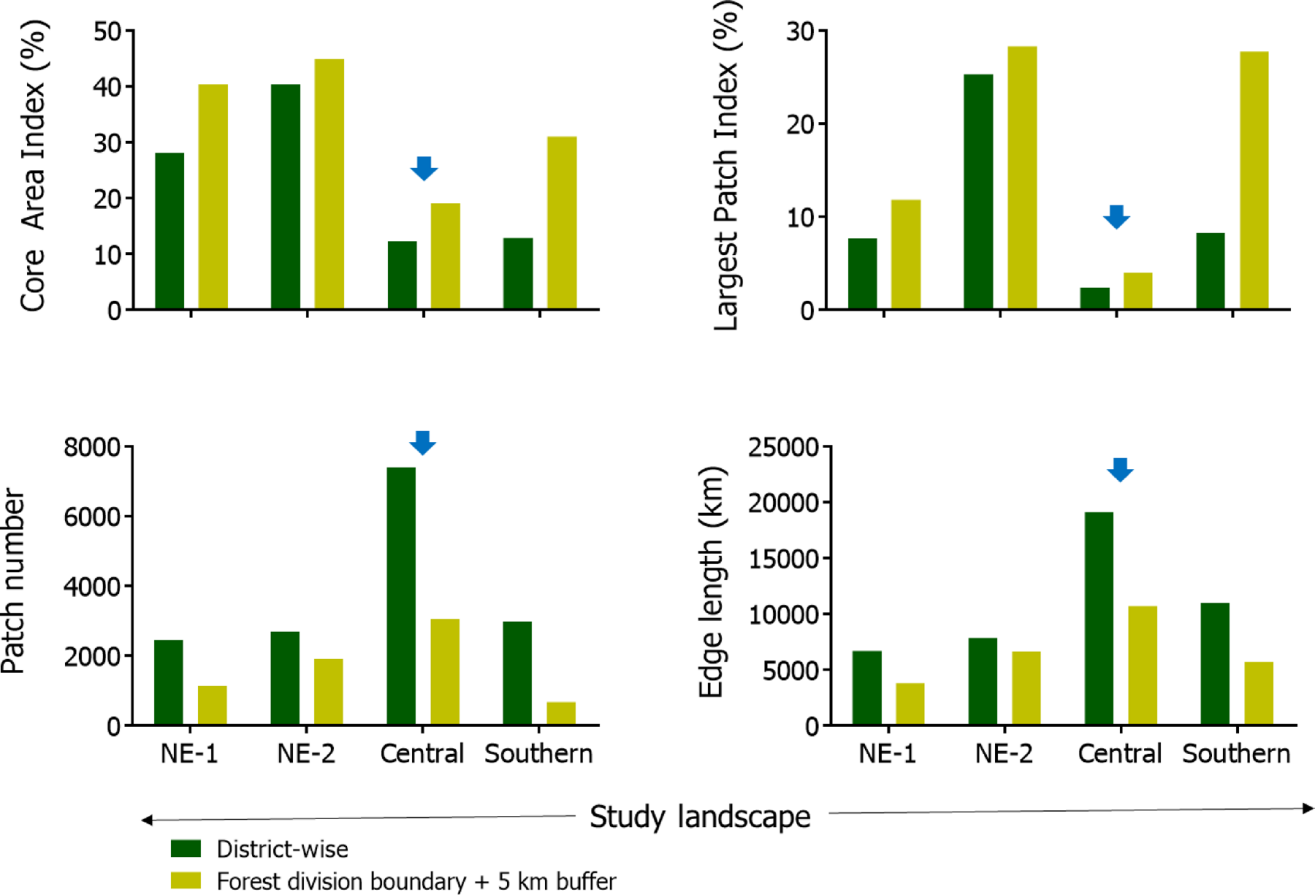
Landscape metrics across the four study landscapes (NE-1, NE-2, Central, and Southern); including core area index (CAI), largest patch index (LPI), patch number (PN), and edge length (EL) (refer to Table S3 for details). Blue arrows indicate that the Central landscape showed the lower LPI and CAI, and the higher EL and PN, suggesting a higher degree of fragmentation.

fGCM levels ranged from 0.05 to 2.42 µg/g, with an overall mean of 0.60 µg/g, while fT3 levels ranged from 0.13 to 1.00 µg/g, with an overall mean of 0.37 µg/g in free-ranging Asian elephants living in human-production landscapes. Central populations had significantly higher fGCM (0.81 ± 0. 37 µg/g) and lower fT3 (0.26 ± 0.06 µg/g) levels as compared to the other populations (Fig. 1). Similarly, the overall assessment of landscape metrics indicated the Central landscape to be the most fragmented habitat when compared to Northeast-1 (NE-1), Northeast-2 (NE-2) and Southern landscapes (Figs. 2 and S1).

**Table 1:** Variation in fGCM levels in free-ranging Asian elephants based on population and diet. Statistically significant differences and effects are indicated in bold (based on the Generalized Linear Model*; for comparisons of means, refer to table S2).

Predictor variables	Level	fGCM* (*n* = 153)					
		Estimate	± SE	t value	Pr(>|t|)	CI 2.5%	CI 97.5%
	(Intercept)	**-0.92**	**0.23**	**-4.00**	**< 0.001**	**-1.31**	**-0.54**
Population-wise variation	Central versus:						
	North-eastern-1	**-0.34**	**0.13**	**-3.00**	**< 0.01**	**-0.60**	**-0.09**
	North-eastern-2	**-0.84**	**0.12**	**-7.10**	**< 0.0001**	**-1.10**	**-0.61**
	Southern	**-0.40**	**0.17**	**-2.40**	**< 0.05**	**-0.70**	**-0.10**
Dietary effects	Dietary index:						
	Faecal C/N ratio	**0.02**	**0.01**	**3.40**	**< 0.001**	**0.01**	**0.03**

*glm(formula = fGCM ~  Population + faecal C/N ratio, family = Gamma (link = log), data = GC data).

**Table 2:** Variation in fT3 levels in free-ranging Asian elephants based on sex, age-class, population, and diet. Statistically significant differences and effects are indicated in bold (based on the Generalized Linear Model*; for comparisons of means, refer to table S2).

Predictor variables	Level	fT3* (*n* = 107)					
		Estimate	± SE	t value	Pr(>|t|)	CI 2.5%	CI 97.5%
	(Intercept)	**-1.60**	**0.16**	**-9.82**	**< 0.0001**	**-1.90**	**-1.26**
Sex-wise variation	Females versus:						
	Males	-0.12	0.07	-1.60	0.11	-0.26	0.03
	Unidentified	0.23	0.22	1.10	0.30	-0.20	0.70
Age-class-wise variation	Adults versus:						
	Sub-adults	**-0.31**	**0.1**	**-3.50**	**< 0.001**	**-0.50**	**-0.13**
Population-wise variation	Central versus:						
	North-eastern-1	**0.44**	**0.10**	**5.20**	**< 0.0001**	**0.30**	**0.61**
	North-eastern-2	**0.50**	**0.10**	**5.73**	**< 0.0001**	**0.32**	**0.70**
Dietary effects	Dietary index:						
	Faecal C/N ratio	**0.01**	**0.004**	**2.24**	**< 0.05**	**0.001**	**0.02**

*glm(formula = fT3 ~ Sex + Age-class + Population + C/N ratio, family = Gamma(link = log), data = T3 data).

### fGCM level across populations

The mean fGCM level significantly varied across the populations with the Central population having the highest levels of fGCM (0.81 ± 0. 37 µg/g; *n* = 52) followed by NE-1 (0.59 ± 0.36 µg/g, *n* = 35), Southern (0.45 ± 0.18 µg/g, *n* = 21) and NE-2 (0.38 ± 0.34 µg/g, *n* = 45) populations (Fig. 1; Table 1 and S2). Even though fGCM levels were significantly different between NE-1 (0.59 ± 0.36 µg/g) and NE-2 (0.38 ± 0.34 µg/g; *P* < 0.01), these levels were not significantly different from those of the Southern populations (0.45 ± 0.18 µg/g; Fig. 1; Table S2).

**Fig. 3:**
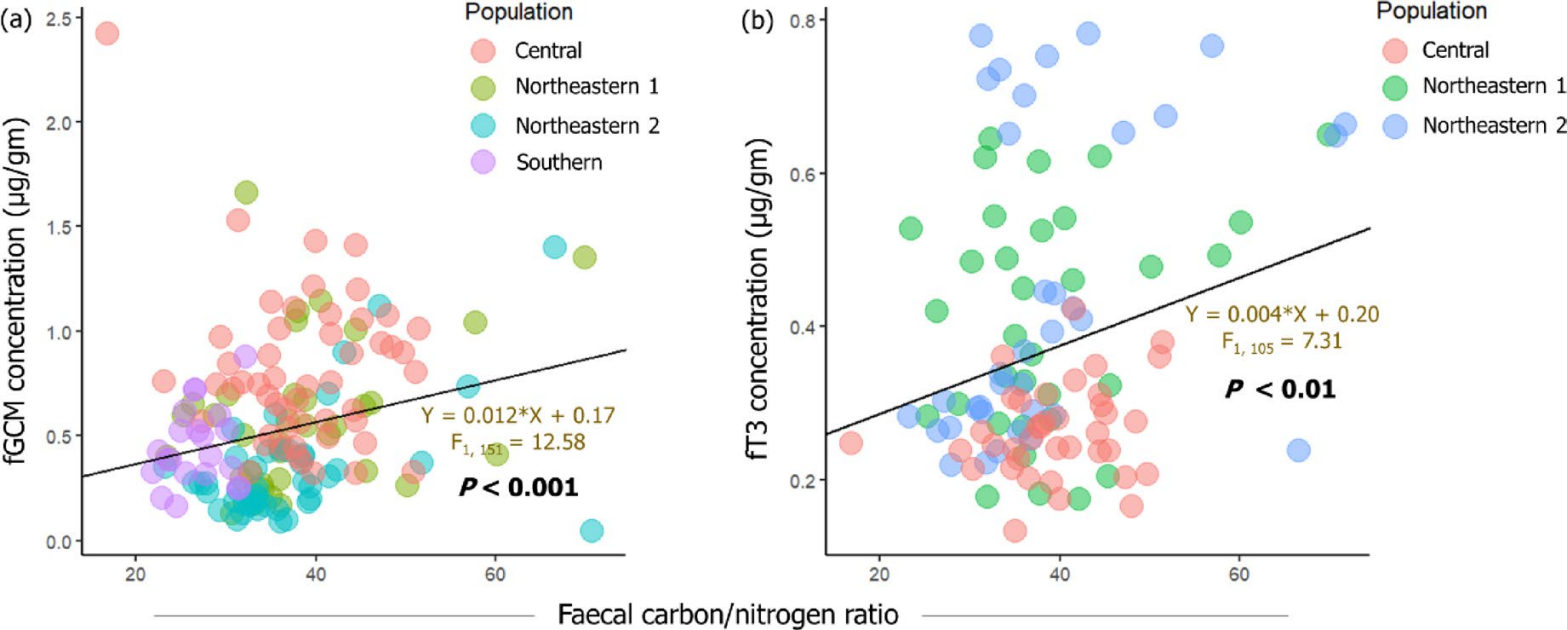
Relationships between faecal C/N ratio and (a) fGCM and (b) fT3 levels in free-ranging Asian elephants across the study landscapes. The scatterplots display significant positive associations, indicating that a decline in dietary quality (reflected by a higher faecal C/N ratio) is associated with higher fGCM and fT3 levels.

Dietary quality was another important predictor, with the faecal C/N ratio significantly influencing fGCM levels (*P* < 0.05; Fig. 3; Table 1). Overall, a significant positive association was observed between fGCM levels and the faecal C/N ratio (*P* < 0.001; Fig. 3; Table 1), suggesting that lower quality of diets is associated with higher levels of fGCM. However, population-level comparisons showed positive associations between the faecal C/N ratio and fGCM levels in NE-1, NE-2 and Southern populations—reaching strong statistical significance only in NE-2—while a moderate negative association in the Central population (*P* < 0.10; Fig. S2). The Southern population had a significantly lower faecal C/ N ratio (26.81 ± 3.13, *P* < 0.001) as compared to the Central (38.51 ± 7.00), NE-1 (38.41 ± 9.72) and NE-2 (37.87 ± 10.64) populations (Fig. S3).

### fT3 levels across populations

In contrast to the levels of fGCM, the mean fT3 level was significantly lower in the Central population (0.26 ± 0.06 µg/g, *n* = 38) as compared to NE-1 (0.41 ± 0.15 µg/g, *n* = 33) and NE-2 (0.45 ± 0.20 µg/g, *n* = 37) populations (Fig. 1; Table 2; Table S1). fT3 levels were not significantly different between NE-1 and NE-2 populations (*P* = 0.97; Fig. 1, Table S1). Overall, faecal C/N ratios were found to be positively associated with fT3 levels (*P* < 0.05) (Fig. 3; Table 2). However, population-level comparisons indicated a significant positive association between faecal C/N ratios only in the NE-2 population (Fig. S2). There were no significant variations in fT3 levels across the sexes (Table 2 and S2). However, adult individuals were seen to have significantly higher fT3 levels (0.39 ± 0.17 µg/g, *n* = 90) as compared to subadult individuals (0.25 ± 0.07 µg/g, *n* = 17; *P* < 0.001) (Table 2 and S2). A Pearson’s correlation test revealed no significant relationship between fT3 and fGCM levels (95% CI [-0.259, 0.119], *P* = 0.46).

### Landscape disturbance metrics

The key landscape metrics, including core area and largest forest patch indices, were lower and, similarly, edge length and patch numbers representing fragmentation were higher in the Central landscape as compared to NE-1, NE-2 and the Southern landscapes (Figs. 2 and S1; Table S3). The forest cover index showed the highest values for the very dense forest type in NE-2 and moderately dense forest type in the Central region (though these are mostly planted forests) (Fig. S1; Table 3 and S3). The NE-1 and NE-2 landscapes had protected areas with NE-2 having the single largest protected area, but none in the Central and Southern Indian landscapes (Table 3). The largest single forest patch covers more than one-fourth of the total landscape area in NE-2 and Southern landscapes, but much less in the other landscapes. Aggressive human responses to elephant depredation of crops were clearly the highest in the Central population where people aggressively used ‘*hula* parties’ to chase elephants (Table S4). Levels of EHN, including unnatural elephant deaths and human fatalities due to elephant attacks, were relatively lowest in the Southern landscape. These metrics indicated that the Central landscape has the highest disturbance as compared to NE-1, NE-2 and Southern landscapes (Table 3).

**Table 3:** Key landscape disturbance metrics representing variations in landscape (fragmentation) parameters, availability of dense forests and protected areas, levels of human aggression and approaches used to deter elephants across the four study populations.

Landscape indices	Study landscape			
	North-eastern-1	North-eastern-2	Central	Southern
Forest cover area (sq km):				
Forest division boundary plus 5 km buffer area (FB5)	1038.0	1521.0	1694.0	1001.0
Key landscape (fragmentation) metrics*:				
Edge length (km)	3806.0	6683.0	10701.0	5729.0
Core area (%)	41.0	45.0	19.0	31.0
Largest patch index (%)	12.0	28.0	4.0	28.0
Very Dense Forest (sq. km)	295.0	429.0	243.0	153.0
Moderately Dense Forest (sq. km)	115.0	326.0	601.0^a^	741.0^a^
Presence of Protected Areas (PAs)	Gorumara National Park (~ 79 sq km) and Chapramari Wildlife Sanctuary (~ 96 sq km)	Buxa Tiger Reserve (~ 760 sq km) and Jaldapara National Park (~ 216 sq km)	No large PAs (some patches of reserve forests, monoculture plantations^a^ under Kharagpur and Medinipur forest divisions)	No major PAs (adjoins some wildlife reserves, forest fragments, coffee and monoculture plantations^a^)
Elephant density (per sq km)^b^	~ 1.0	~ 0.3	~ 0.05	~ 0.15
Human population density (per sq km)^c^	~ 600	~ 500	~ 800	~ 300
Level of human aggression during anti-depredation action	Aggressive	Moderately aggressive	Highly aggressive	Least aggressive
	Controlled disturbance approach by ‘rapid response team’ (RRT); Crowds and large groups of villagers chase and deter elephants through loud noises and throwing stones	Controlled disturbance approach by RRT of the wildlife department using loud noises (drums and firecrackers) to deter and chase elephants	More aggressive approach by ‘hula parties’ using spears and poles (*mashaal*) with fire balls which are often thrown at the elephants (see Table S4)	Chasing by relatively smaller groups of people, prevention using electric fences and RRT involvement
Unnatural elephant deaths (per annum)^d^	~ 3.0	~ 2.0	~ 5.0	~ 2.0
Human deaths due to elephant attacks (per annum)^d^	~ 17.0	~ 16.0	~ 20.0	~ 3.0

^a^Includes open forests with monoculture plantations of exotic species (non-forage species for elephants such as Acacia, teak and Eucalyptus);^b^*MoEF&CC*^30^; ^*c*^Census 2011^71^; ^d^Mortality data were normalized per year based on the statistics presented (for the study sites within four landscapes) in Majumdar^32^ and KFD report^72^. *Refer to Table S3 for detailed landscape metrics.

## Discussion

The stress-related physiological and metabolic states of free-ranging Asian elephants differ across human-production landscapes with varying levels of fragmentation and anthropogenic disturbance. We found that elephants from the Central Indian population had higher fGCM and lower fT3, as compared to populations elsewhere, potentially indicating a compromised health status for elephants living in highly fragmented habitats and experiencing much higher levels of anthropogenic stress. Our earlier research in a Southern population^5^ showed that lower fGCM levels were linked to superior dietary quality (indicated by lower faecal C/N ratios) in crop-foraging elephants; we observed a similar relationship in the NE-1 and NE-2 populations, but the Central population deviated from this pattern. This implies that severe anthropogenic disturbance, rather than dietary quality, may have a stronger influence in stress physiological responses among elephants.

Various contexts such as elephant social structure, seasonality, dietary quality, health and reproductive status have been shown to influence the physiological state in elephants^5,8,33–35^although their long-term consequences on overall fitness remain unexplored. However, anthropogenic disturbances are often significantly associated with higher fGCM levels (~ 80% of studies showing consistently higher) in both Asian and African elephants (reviewed in Pokharel and Brown^8^). In a similar context, elephants ranging within a human-production landscape with relatively low levels of anthropogenic stressors compared to other such landscapes in India showed lower levels of fGCM than those ranging within protected areas^5^. This reduction in fGCM levels was attributed to nutritional benefits gained from cultivated crops which presumably lowered their physiological stress^5^.

The degree of anthropogenic disturbances, associated-management practices and their implementation vary across populations. Therefore, given the distinctive nature of landscapes and the responses of elephants, it is essential to exercise caution when attempting to extrapolate stress patterns from one landscape to another. Importantly, elephants’ individual experiences, personality traits, and contexts determine how they respond to certain stressors^8^. Evidently, a positive correlation has been observed between anthropogenic disturbances and stress response in free-ranging Asian elephants, be it while crossing roads or chasing operations aimed at reducing elephant presence near settlements, agricultural fields, or tea plantations^36–38^. Therefore, higher fGCM levels observed in the Central populations could be due to a higher degree of habitat fragmentation and associated anthropogenic disturbance observed in this landscape. As samples from all four landscapes were collected during the peak crop-foraging period, coinciding with agricultural activities such as crop harvesting, both farmers and wildlife staff were actively engaged in crop protection efforts, including chasing operations. However, the frequency and intensity of chasing operations, such as deploying the so-called ‘*hula*’ party, in which villagers, use flaming torches and spears to deter elephants, often causing serious injuries to the elephants (see Table S4), were remarkably higher and primarily confined to the Central Indian landscape. Such intense conflict management practices may have heightened stress reactivity in elephants, leading to higher fGCM levels.

Considering the high-risk and the high-gain strategy behind crop foraging^39^earlier research on free-ranging Asian elephants indicated a positive association between fGCM levels and faecal C/N ratios, implying nutritional benefits obtained from crop foraging were potentially pacifying the stress reactivity in a Southern Indian population with relatively lower risk or disturbance^5^. Similar trends in food availability, dietary quality and nutrition lowering stress levels have been observed in other mammalian species^40–42^. However, a negative association observed between faecal C/N ratio and fGCM levels in elephants within the Central population suggests that stress due to anthropogenic disturbance (higher risk) may have had a more pronounced effect on the HPA axis than the nutritional benefits derived from crop foraging (lower gain). Thus, the synergistic effects of habitat fragmentation, leading to extreme EHN, along with associated management practices and foraging-related/nutritional challenges, may have contributed to higher fGCM levels in elephants within the Central population. The NE-1 population showed significantly higher fGCM levels compared to NE-2, likely due to relatively high fragmentation and disturbance in NE-1 where elephants were observed being chased from one plantation to another, unlike in NE-2.

THs, particularly T3, play a major role in regulating energy homeostasis by altering the cellular metabolic rate and energy expenditure, producing heat and even modulating the expression of glucose metabolism^43^. Maintaining normal thyroid function is vital for sustaining regular reproductive, metabolic, and growth processes; any perturbations in thyroid levels may lead to compromised biological functions^44,45^. Studies have shown that lower T3 levels are linked to energy-restricted phases, resulting in a reduced metabolic rate during resource-deficient periods in different species^15,21^. Elephants and other free-ranging animals have received relatively little research attention concerning thyroid hormones, probably due to difficulty in collecting serum samples for analyses (Table S1). While most studies focus on serum collected from captive elephants to assess the effects of reproductive stages, health or gut microbiomes on THs, some studies have used non-invasive techniques in analyses of fT3^19,20,25–29^ (Table S1). One such study in free-ranging African elephants reported that the increased availability of resources and higher temperature lowered fT3 levels^19^. Although more studies are warranted in both captive and free-ranging elephants, it appears that changes in T3 levels are primarily linked to energy-homeostasis-based events, with higher T3 levels reflecting a more active metabolic state, often being associated with abundant resources and less related to reproductive events.

Nonetheless, the association between disturbance contexts and the HPT axis in elephants is not well known. Studies on ungulates show no correlation between fT3 and anthropogenic disturbances, except in one study where disturbance influenced fT3 when temperature was accounted for (reviewed in Pasciu et al.^46^). However, species-specific differences in thyroid stimulation and reactivity cannot be overlooked. Lower fT3 levels observed in elephants from the Central population could potentially reflect hypometabolism to conserve energy under challenging situations. During our fieldwork, we observed that the limited availability of intact or large forest patches influenced the foraging behaviour of elephants in the Central population, as they were constantly driven by villagers from one patch (typically monoculture plantation) to another. As a result, these elephants had minimal opportunities for resting and foraging, often resorting to quick foraging sessions in crop fields during late night or early hours of the day when human activity was at its lowest. This pattern was not apparent in NE-1 and NE-2 populations, where the presence of intact or large forest patches allowed elephants to take refuge and rest during the day, and forage crops more extensively at night.

We propose a possible mechanism to explain the condition of reduced metabolism or hypometabolism in elephants. The ‘abnormal foraging effect’ hypothesis may explain the effects of severe anthropogenic disturbances that disrupt the normal foraging behaviour in elephants (leading to nonselective foraging, altered foraging timing or rapid foraging to avoid humans) when in human-production landscapes, suppressing the HPT axis (equally affecting the HPA axis). This may be similar to endocrinopathies observed in humans with altered eating habits^47^. The positive association observed between fT3 and faecal C/N ratios appears to be primarily driven by faecal carbon content rather than nitrogen (Fig. S4), probably indicating that higher thyroid activity accelerates gut motility and basal metabolic rate, which may reduce nutrient absorption efficiency, especially complex carbohydrates and fibers, leading to more carbon excreted in faeces^48^. However, in the absence of detailed thyroid-related research on free-ranging elephants, we can only speculate that thyroid levels may be modulated by several factors including dietary intake of essential micronutrients and trace elements^49,50^varying metabolic demands across age and sex-classes^51–53^reproductive state, and seasonality and resource distribution.

Our study underscores the importance of integrating physiological dimensions into conservation planning in the context of elephants living in human-production landscapes. While these landscapes provide access to superior nutrition and are considered as prime habitats^54,55^it is apparent that elephants here experience complex physiological costs. It is important, therefore, to monitor physiological markers for evaluating the effects of different anti-depredation practices employed in conflict mitigation. One should obviously exercise caution while interpreting data from fGCM and fT3, as these levels may signify physiological adjustments in elephants with varied consequences for their fitness^8,56^. This study highlights the need for longitudinal evaluation of various physiological markers across diverse contexts to deepen our understanding of how elephants adapt to diverse landscapes with varying levels of anthropogenic stressors, and how these adaptations affect their overall survival in the long term.

## Methods

### Study sites

**Fig. 4:**
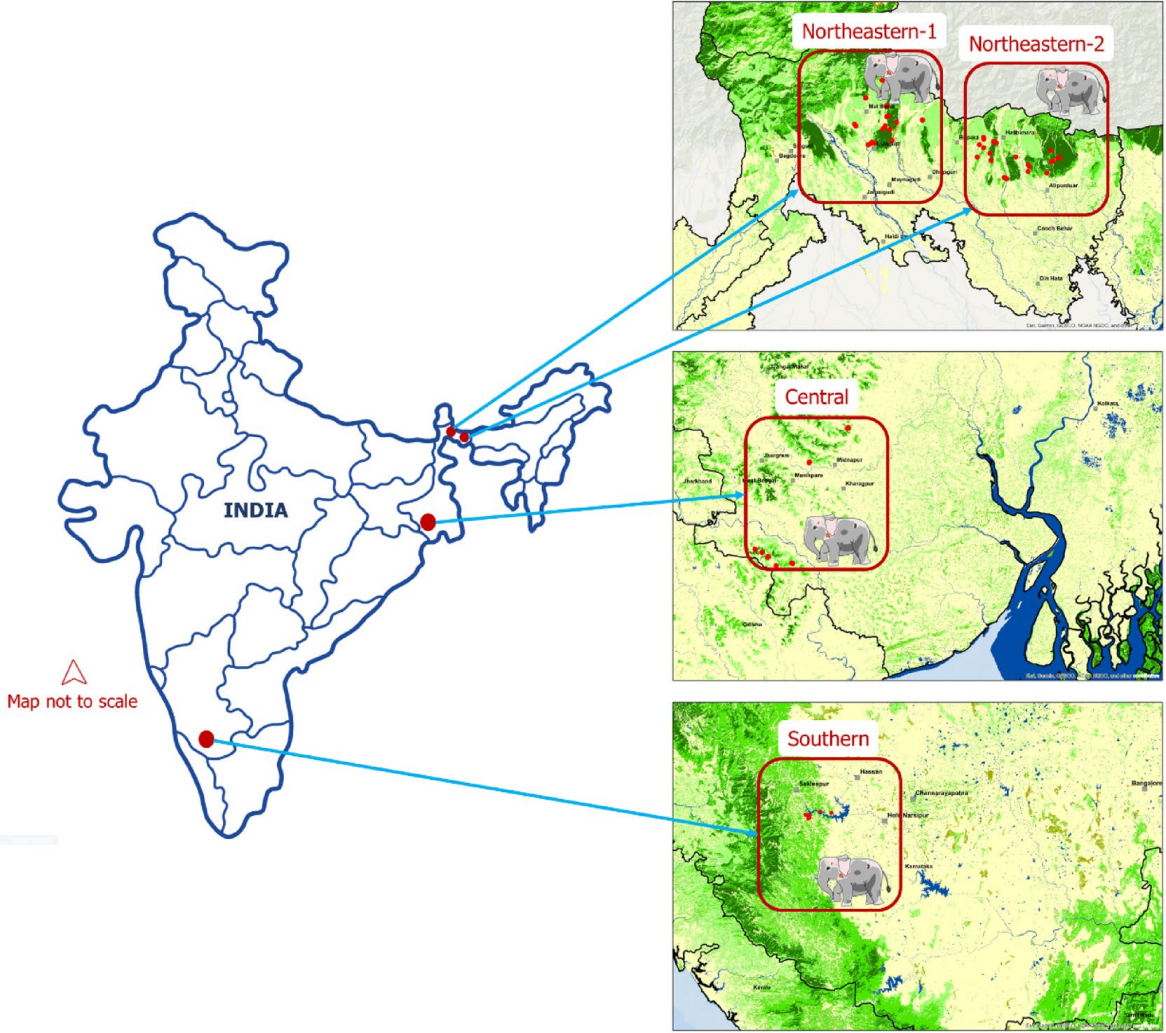
Map of India (not to scale) showing the study populations of free-ranging Asian elephants, including Northeastern (NE-1 and NE-2), Central and Southern populations. GPS coordinates of faecal sampling locations are represented as red dots within the inset maps.

We selected two elephant populations from the Northeastern and one population from the Central landscape of West Bengal state, India, for fresh faecal sampling and comparison with a Southern population of Karnataka state (Fig. 4; henceforth, the term “population” is used in a broad sense to refer to the elephants in a given geographical entity (NE-1, NE-2, Central and Southern) sampled, while “landscape” refers to the corresponding geographical habitat i.e., NE-1, NE-2, Central and Southern, sampled).

#### (i) Northeastern India study population (NE-1 and NE-2)

Spread along the foothills of the Eastern Himalaya, the northern West Bengal (26° 68´– 27° 11´ N to 89° 86´ – 87° 99´ E; Fig. 4, Fig. S1) landscape experiences high levels of EHN. From this landscape, we selected: (i) the more fragmented Jalpaiguri/Gorumara National Park (henceforth, Northeast-1 population or NE-1), and (ii) the less fragmented Buxa/Jaldapara National Parks (henceforth, Northeast-2 population or NE-2) for further sampling (for details on EHN, elephant and human densities, refer to Table 3).

#### (ii) Central India study population

The southern West Bengal (22°0´ – 24°0´ N to 85°30´– 88°30´ E; Fig. 2; Fig. S1) is a part of the highly fragmented Central Indian elephant habitat with very high levels of EHN (refer to Table 3 for details on EHN, elephant and human densities). Since 1986-87, a clan of “migratory” elephants from the states of Jharkhand and Odisha has been ranging over several districts in southern West Bengal with the numbers growing over time and several elephants now becoming “resident” here^30,57^. The lack of natural (as opposed to plantations) and contiguous forest patches, and expansive areas of agricultural fields have led to frequent and intense EHN in this landscape, with elephants foraging on crops and taking refuge in the plantations. We selected areas in the Medinipur and Kharagpur districts of southern West Bengal for sampling (Fig. 4, Fig. S1). Human responses in this landscape included extremely aggressive practices, such as deploying ‘*hula* parties’ or groups of 15 to 30 people using flaming torches or iron rods/spears, to drive away elephants from villages or plantations (Table S4).

#### (iii) Southern India study population

This study population represented randomly selected season-matched data from previously published research^5^ in Hassan district, Karnataka (13°00´ N to 76°6´ E; nearly 70–80 elephants ranging in ~ 300 sq km of agricultural field, coffee plantation and fragmented forested areas in the Western Ghats; Fig. 4, Fig. S1; Table 3).

These four landscapes represent varying levels of fragmentation, EHN (as indicated by the number of unnatural elephant deaths and human deaths due to elephant attacks) and anthropogenic disturbances, with the NE-1, NE-2 and Central landscapes having relatively higher levels of EHN than the southern landscape, and the Central landscape witnessing the most aggressive human response towards elephants (see Table 3).

### Faecal sample collection

Fieldwork was conducted only during the wet season (November and December 2018: months corresponding to the crop harvesting period) to avoid seasonal variability^33^. All samples were collected opportunistically from crop-foraging sites in the landscape, aided by information from local communities and forest department officials about crop-foraging events and locations of the elephants. As these incidents habitually occurred at night, we conducted our fieldwork during the events (at night itself) or shortly after, at the earliest possible time of the day, allowing us to collect fresh faecal samples from the crop-foraging elephants. To determine the timing of defecation, we either inquired farmers or assessed the freshness of the dung by checking for surface mucous or moisture on the dung boli. We collected samples no older than about six hours post-defecation to prevent bacterial degradation on hormone metabolites^58^. We classified the age of each sampled individual as: adult (over 15 years), subadult (5 to 15 years), juvenile (1 to 5 years), and calf (under 1 year). For direct sightings, we used the relative shoulder height method^59^ and if not, we used the dung bolus circumference method for age estimation^33,60^. At most field sites, we primarily collected faecal samples from adult and subadult individuals. The sex of the elephants was determined through direct sightings, inquiries with farmers, or opportunistic photos and videos taken when elephants cross the roads and plantations or while crop-foraging. Samples for which we could not identify the sex were marked as ‘unidentified’ (Table S5). To minimize duplicate sampling, we collected samples over a short time frame from widely spaced locations (~ 80 to 100 km for NE-1 and NE-2, and 700 to 800 km for the Central population), maximizing our daily field travel distance.

Samples were stored in a portable Coleman Xtreme cooler box, maintaining a temperature of around − 20 °C in the field, aided by the lower ambient temperature. The frozen samples were later transferred to a -20 °C refrigerator until analysis, preventing degradation of target hormones and steroids^13^. We collected a total of 156 fresh faecal samples from the NE-1, NE-2 and Central populations (refer to Table S5 for detailed sample distribution). Due to very few samples collected for calves and juveniles in NE-1 and NE-2, our analyses were restricted to individuals aged above c.5 years (including subadults and adults). Thus, out of 153 samples, 107 were analyzed for fT3, and 153 were analyzed for fGCM, including 21 previously analyzed samples from adult individuals of the Southern population^5^.

### fGCM and fT3 extraction and analyses

We followed the protocols described in Ganswindt et al.^61^ and Pokharel et al.^33^ to extract faecal steroids and analyze immunoreactive fGCM. For measuring the fGCM levels, we used the group-specific 11-oxoetiocholanolone enzyme immunoassay or EIA (lab-code: 72T with an antibody produced against 5β-androstane-3α-ol-11-one-17CMO: BSA and 5β-androstane-3α-ol-11,17-dione-17-CMO-biotinyl-3,6,9-trioxaundecanediamine as a label; Prof. Rupert Palme Laboratory, University of Veterinary Medicine, Vienna), which has been well-validated for free-ranging Asian elephants^5,33,34^. The assays were analytically validated by demonstrating parallelism between the serial dilutions of faecal extracts and the standards (Fig. S5 a and c). Faecal extracts were diluted at ratios of 1:10 and 1:20. The assay sensitivity was 0.011 µg/g of faecal dry weight (at 90% binding). The intra- and inter-assay coefficients of variations were 5.4% and 9.1%, respectively.

For measuring immunoreactive fT3, we used the same faecal extracts prepared for the fGCM analysis. We used the commercially available T3 EIA kit (catalog #K056; Arbor Assays, Ann Arbor, MI with a DetectX^®^ Triiodothyronine (T3) antibody, a sheep antibody specific for Triiodothyronine, and DetectX^®^ Triiodothyronine (T3) Conjugate as a label) which has been well validated and proven effective in measuring fT3 levels in both free-ranging African^19^ and Asian elephants (captive and free-ranging^20,26^). The assays were analytically validated by demonstrating parallelism between the serial dilutions of faecal extracts and the standards (Fig. S5 b and d). The intra- and inter-assay coefficients of variations were 7.1% and 10.2%, respectively. Faecal dilutions of 1:25, and in some cases 1:50, were used for fT3 analyses (Fig. S6).

### Dietary quality

The faecal C/N ratio is widely used as a non-invasive proxy of dietary protein availability and overall dietary quality in mammalian herbivores^62,63^. A high faecal C/N ratio, meaning low faecal nitrogen content, typically indicates a diet of poorer quality^63^. Elephants having access to agricultural land are known to choose nutritively richer and more palatable crops over wild forage plants^54,64^. Furthermore, cultivated cereal crops were shown to provide significantly more protein, calcium and sodium than the analogous wild grasses^54^. Although dietary quality as measured by faecal C/N ratio has been reported to possibly regulate the physiological state in Asian elephants^5^other nutritional components, such as carbohydrates, lipids and minerals, are equally important. We, therefore, quantified faecal C: N, as a surrogate of the crude protein content by measuring total carbon and total nitrogen contents in dried-homogenously powdered faecal samples combusted in an automated TruSpec Micro Series CN Analyzer^5^.

### Assessment of landscape disturbance metrics

For species such as elephants which typically require large land area for movement, resources, and reproduction, quantifying habitat connectivity or fragmentation provides a measure of anthropogenic disturbances^65^. Landscape disturbance metrics were used to characterize variation in fragmentation and anthropogenic disturbances across the four landscapes. For this purpose, we downloaded the forest cover data from the Forest Survey of India^66^ which categorizes forest cover as very dense, moderately dense, open and scrub forest types based on the percentage of canopy density (Table S3). We used the software Fragstats 4.2, a spatial pattern analysis programme that quantifies the spatial heterogeneity of the landscape^67,68^to measure the landscape metrics, focusing mainly on area, edge and aggregation metrics such as edge length (EL; the perimeter of a patch or the forest cover), edge density (ED; the total number of edges divided by the total area of the landscape), patch numbers, patch density (PD; the number of patches over the total area under forest cover), core area (CAI; the percentage of core area to the patch area) and largest patch (LPI; the percentage of the largest forest patch area in relation to the total area of the landscape) indices^67,68^to understand the degree of fragmentation across study sites (Table S3). We quantified landscape metrics based on administrative boundaries: (i) the total district-wise boundary (DB) and (ii) the forest division boundary with an additional 5-km buffer (FB5) where crop raiding elephants are most likely to forage (Fig. S1; Table S3).

Alongside landscape metrics, we included the presence of Protected Areas (PA), human population density, and details on EHN. We qualitatively defined the levels of human aggression toward elephants during anti-depredation action, based on our field observations, discussions with local stakeholders and media reports, to provide the extent of disturbance across the four study landscapes, collectively referred to as ‘landscape disturbance metrics’ (Table 3).

### Statistical analyses

We selected four predictors for our analysis, comprising the categorical predictors: age, sex, population, and a continuous predictor, faecal C/N ratios associated with diet quality, against the continuous positive response variables, fT3 and fGCM (Tables 1 and 2). We performed the analyses separately for fT3 and fGCM (since there was no fT3 data from the southern population). We used the generalized linear model (GLM) using the function ‘glm’ with Gamma-distributed errors and *log* as a link function to predict the effects of predictors on fT3 and fGCM. To evaluate the influence of predictors and interaction terms, we generated a set of GLMs derived from a saturated global model that included four predictors and all possible interaction terms, using the *dredge* function in the package ‘MuMIn’ (Tables S6 and S7). We report only the top five models based on the lowest Akaike’s Information Criterion (AIC) and delta AIC scores, and the highest AIC weight (as described in Burnham & Anderson^69^; Tables S6 and S7). The top models identified faecal C/N ratio and population as the only significant predictors of fGCM, while faecal C/N ratio, population, age and sex predictors for fT3 (Tables S6 and S7). Interaction terms did not show any significant effects and were not retained in the final models. Based on the top selected final model, we performed GLMs to evaluate the statistical significance of each predictor, reporting p-values (denoted as *P*) and 95% confidence intervals (using the *confint* function; Tables 1 and 2). We provide the pairwise means across populations for fGCM levels, and across population, sex and age categories for fT3 levels using the *emmeans* function from the ‘emmeans’ package (Estimated Marginal Means), with a 95% confidence level, where *P*-values were adjusted using the Tukey method for multiple comparisons (Table S2). For landscape metrics represented by single data points per index, a simple descriptive approach was employed to evaluate and compare the differences across landscapes (Figs. 2 and S1; Table S3). We used R version 4.3.2 for all analyses^70^. Values of fGCM and fT3 are reported as ‘mean ± standard deviation’ throughout.

## Electronic supplementary material

Below is the link to the electronic supplementary material.

Supplementary Material 1

## Data Availability

Supporting data are available as Supplementary information.
