# Supplementary material for: Physiological responses in free-ranging Asian elephant populations living across human-production landscapes
Source: Sci Rep. 2025 Sep 4;15:32365. doi: 10.1038/s41598-025-06243-y (PMC12411615; doi:10.1038/s41598-025-06243-y)
Supplement: Supplementary file 1 — Supplementary Material 1 [file 41598_2025_6243_MOESM1_ESM.docx]

**Physiological responses in free-ranging Asian elephant populations living across human-production landscapes.**

Sanjeeta Sharma Pokharel^1,2,3, *^, Amir Kumar Chettri^5^, Sunipa Chatterjee^1^, Polani B. Seshagiri^4^, Raman Sukumar^1, *^

^1^Centre for Ecological Sciences, Indian Institute of Science, Bangalore, India

^2^Asian and African Area Studies, Kyoto University, Kyoto, Japan (Current affiliation)

^3^The Hakubi Center for Advanced Research, Kyoto University, Kyoto, Japan (Current affiliation)

^4^Department of Developmental Biology and Genetics, Indian Institute of Science, Bangalore, India

^5^The Co-existence Project, West Bengal, India

*Corresponding authors (SSP and RS)

**Supplementary Information:**


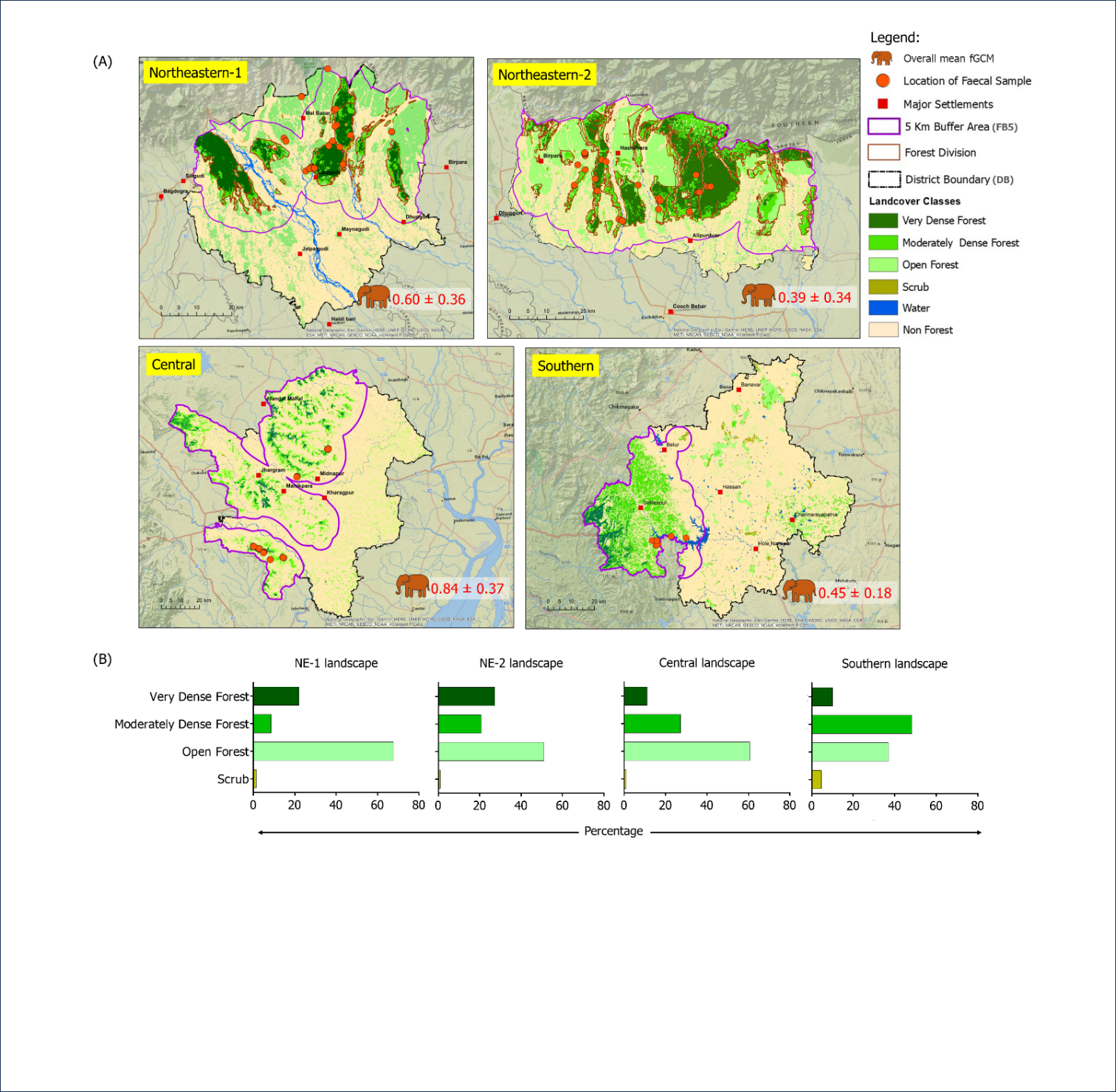


**Figure S1.** (A) Forest-cover across four study landscapes* (NE-1, NE-2, Central and Southern). Forest cover maps show continuous forest cover (dark green), open forest/tea gardens/plantation (light green), agricultural land (yellow), water cover (blue), major human-settlements (red squares), GPS locations of samples (brown dots) and overall mean levels (including all age classes) of fGCM in free-ranging Asian elephants. (B) Bar graphs represent percentage of types of forests across the landscapes.

* The ArcGIS 10.8 software and the forest cover raster data from the Forest Survey of India (2018) as a base map (the boundary shapefiles were downloaded from the respective forest departments of Karnataka and West Bengal) were used to plot the details in the maps. Villages/ areas sampled: (i) in NE-1 (mainly areas around Gorumara and Jalpaiguri): Ramsai, Chapramari, Colakhawa, Budikup, Neora, Telabadi, Sepchu, Khuniya, Kelabari, Panijhora, Lakipada, Sitbari, Bedgudi, Latagudi and Kumarpada), (ii) in NE-2 (includes villages around Jaldapara and Buxa): Jaldapara eastern forest range, Sisamari, Paschim madarihaat, Purba madarihaat, Lankapara, Torsa, Totopara, Uttarrangali banzar, Islamabad, Dumsipara, Rajabhatkhawa, Satali and Nimti, (iii) in Central (Medinipur and Kharagpur): Arabari, Chandrakona, Nayabasat, Godapiasal, Bhadutala, Chandra, Pirakata, Lalgarh of Medinipur and Chandabila, Kasiyabati, Nayagram, Junglekhas, Kesorekha mauja, Kalaikunda mauja, and Jhotiyahati of Kharagpur and areas adjoining Jhargram and (iv) for Southern (Hassan, Karnataka): data was taken from Pokharel et al.^6^. Distinctly visible/identifiable crop remains in faeces included paddy (*Oryza sativa*) and banana (*Musa* species), along with other non-crop fruit remains, such as hog plum (*Spondias mombin*) and Elephant apple (*Dillenia indica*); in addition, some of the samples also had plastic remains (n = 8).


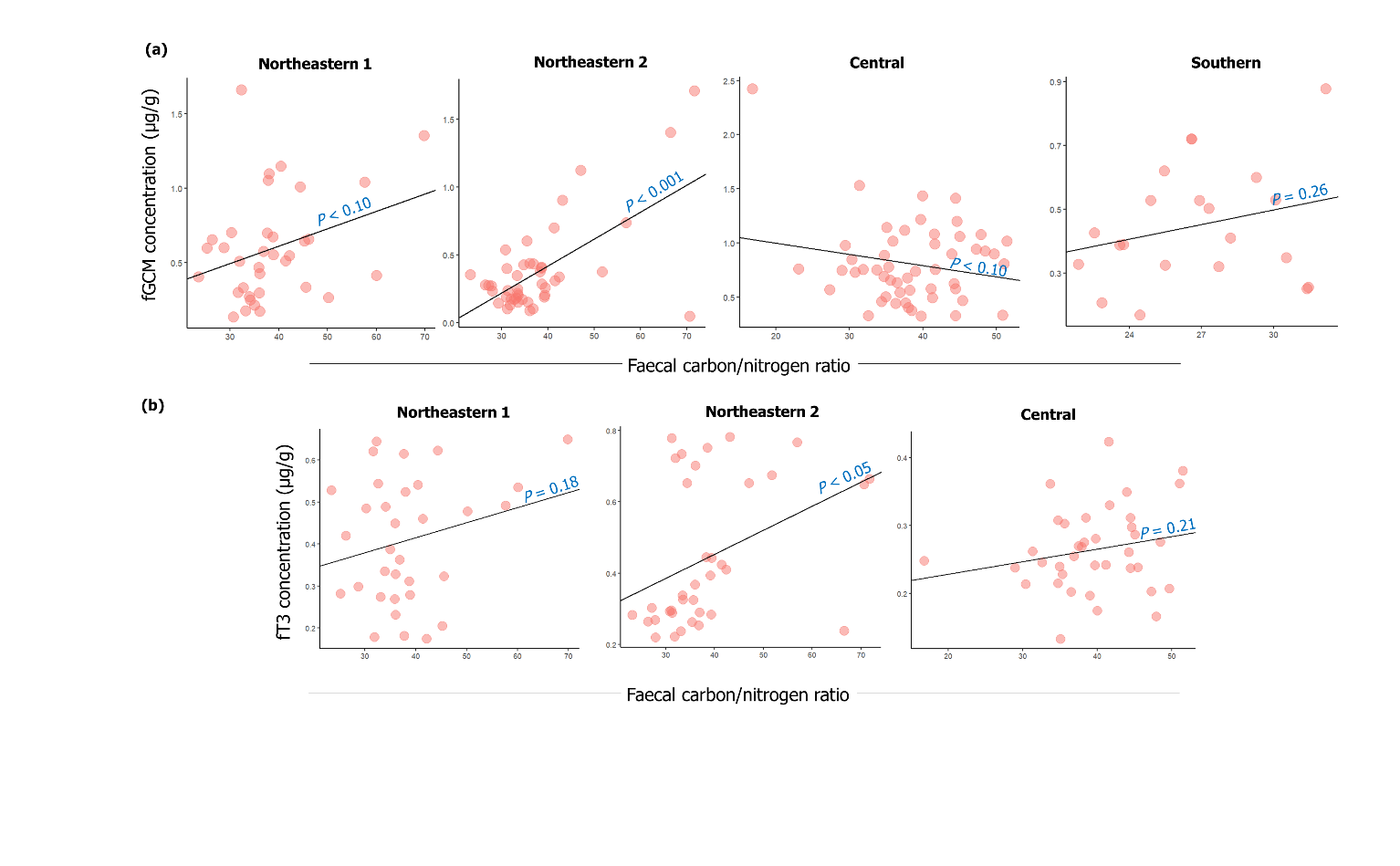


**Figure S2.** Scatterplots show relationships between faecal C/N ratios and (a) fGCM and (b) fT3 levels in free-ranging Asian elephants living across the study populations. Positive correlation trends were observed in NE-1, NE-2 and Southern populations, although some of these associations were not statistically significant, suggesting poor-quality diets associated with elevated fGCM and fT3 levels. In contrast, a moderately negative correlation was observed between fGCM levels and faecal C/N ratios in the Central landscape.


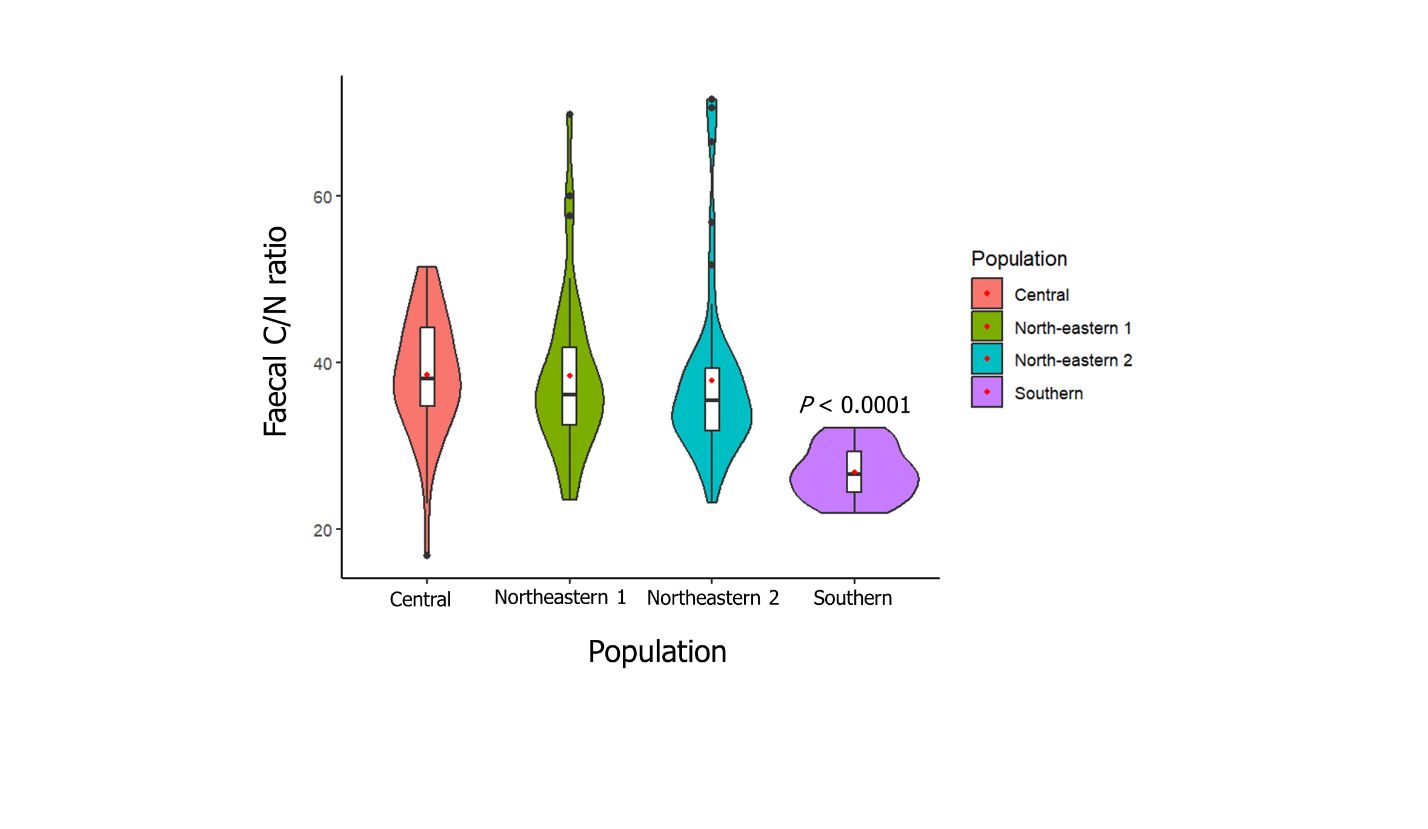


**Figure S3.** Population-level variations in faecal carbon and nitrogen ratios among free-ranging Asian elephants across human-production landscapes. Violin plots depict the distribution of faecal C/N ratios in elephants from the Central, Northeastern (NE-1 and NE-2), and Southern populations. The Southern population had significantly lower C/N ratios (relatively good quality of diets) compared to the others. Boxplots with the violins display the median (with the mean as a red dot) and the interquartile range. The shaded areas of the violin plots represent the smoothed distribution of values based on a probability density function. Statistical significance (p-values) was calculated using the GLM.


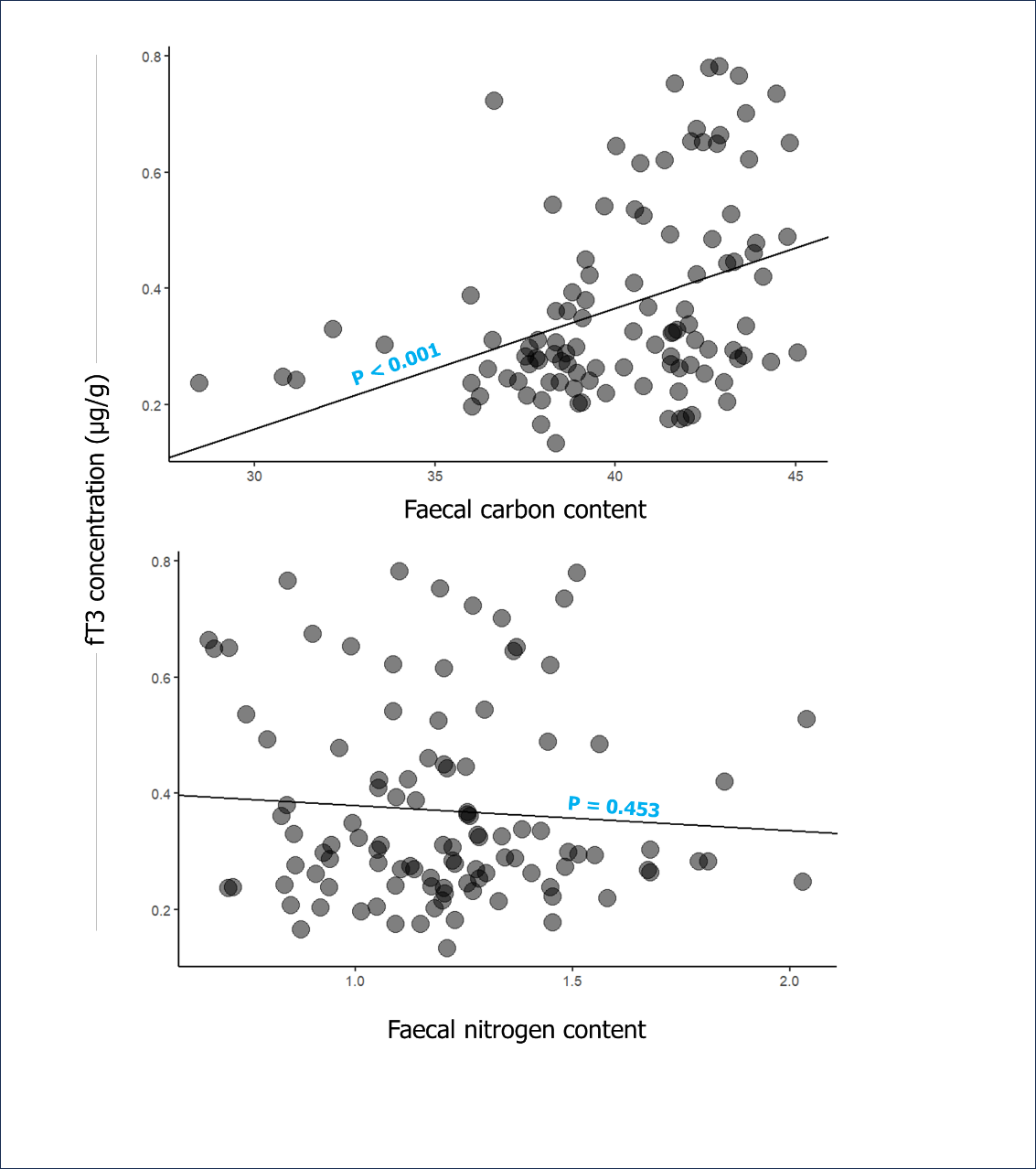


**Figure S4.** Relationships between fT3, faecal carbon and nitrogen contents. A strong positive relationship between faecal C/N ratio and fT3 is driven by faecal carbon content, rather than the dietary protein reflected by faecal nitrogen content.


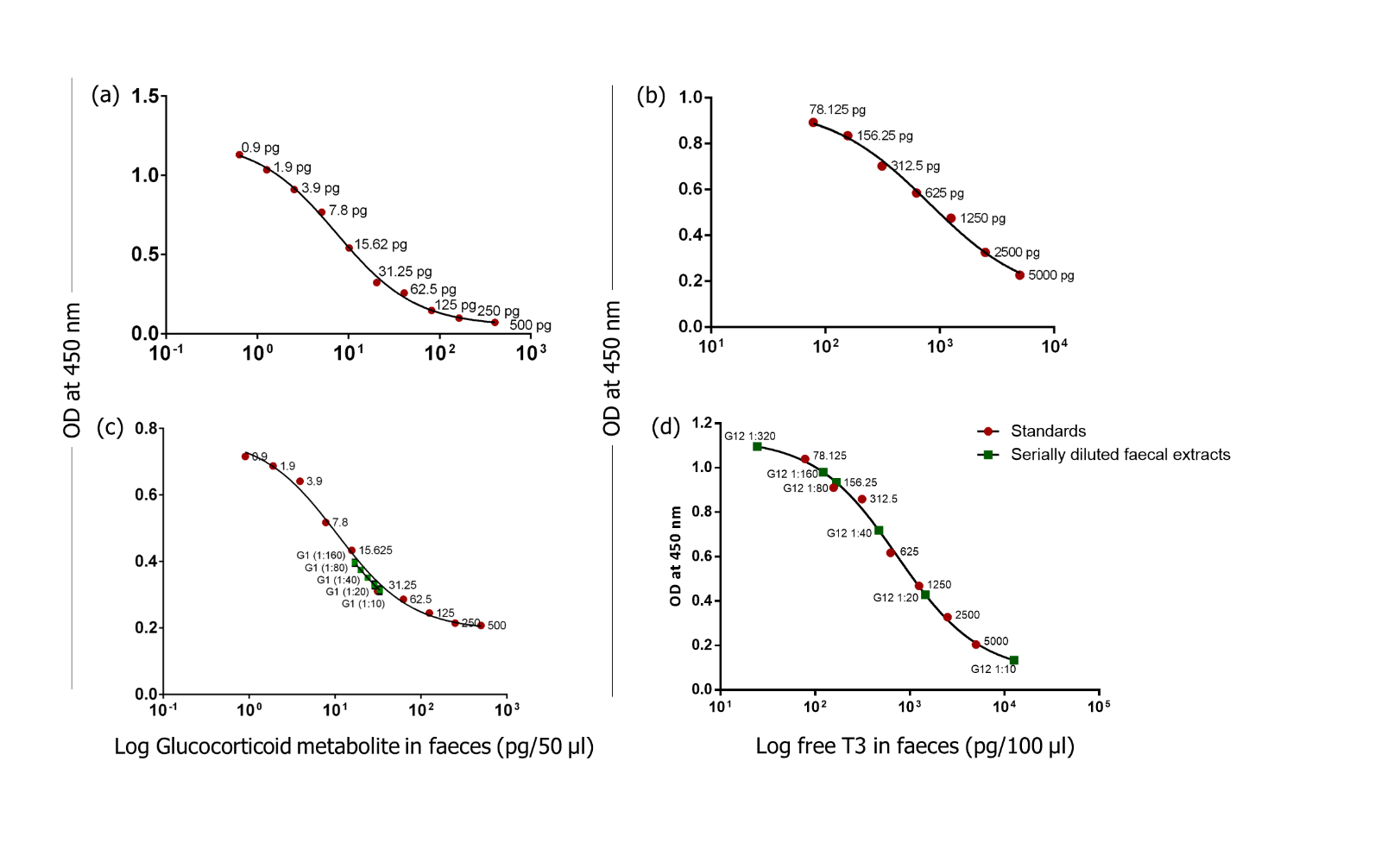


**Figure S5.** Standard curves (a, b), along with parallelism (c, d) between serial diluted faecal extracts and standards, obtained for (a, c) 11-oxo-etiocholanolone (72T assay; Prof. Rupert Palme Laboratory, University of Veterinary Medicine, Vienna) and (b, d) total triiodothyronine (K056 EIA, Arbor Assays) assays based on optical densities measured at 450 nm.


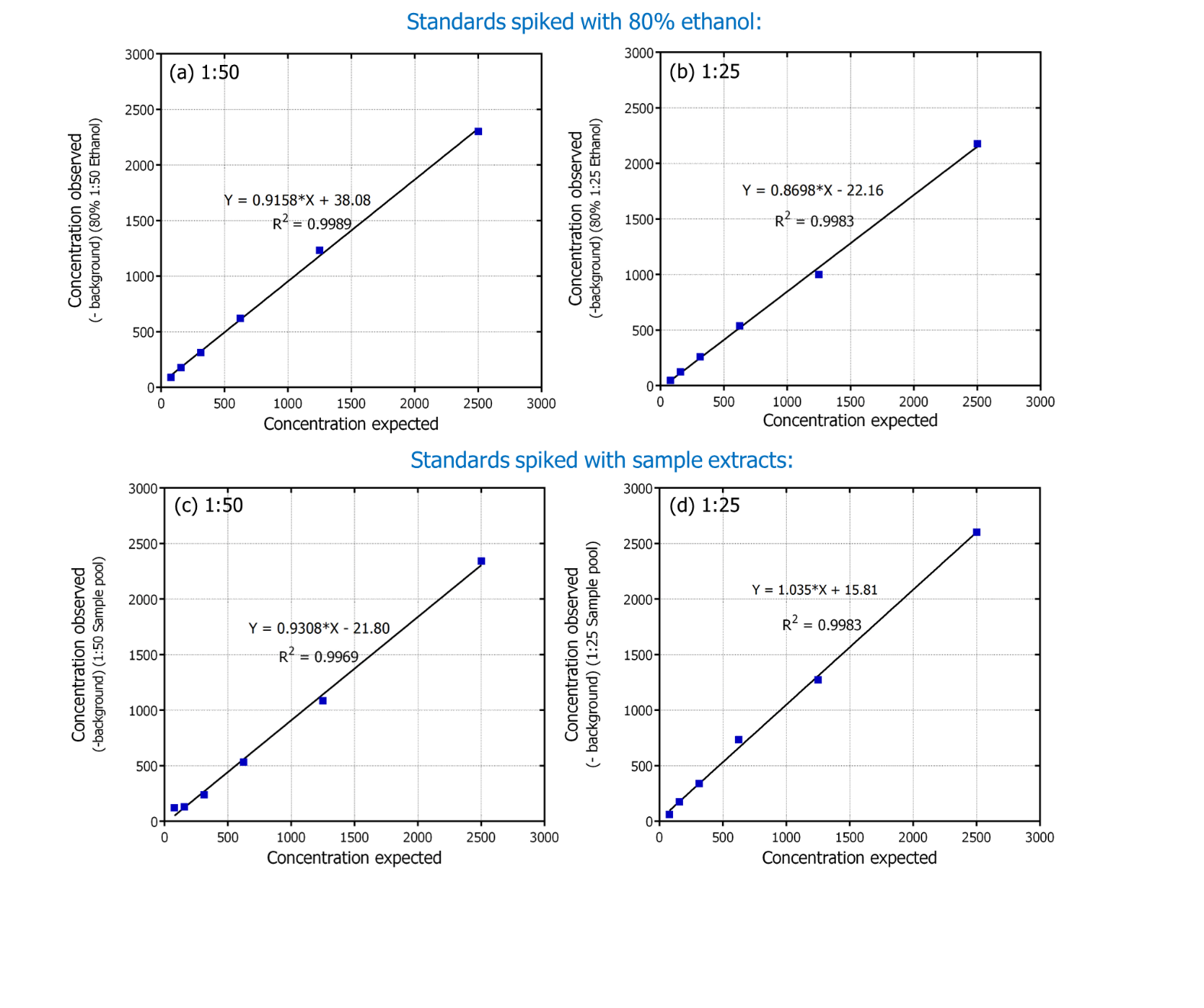


**Figure S6.** Effect of types of diluents (80% ethanol and faecal extracts in assay buffer) on accuracy of fT3 assay. Observed versus expected concentrations of serially diluted total triiodothyronine standards in (a) 1: 50 and (b) 1:25 of 80% ethanol, and (c) 1:50 and (d) 1:25 faecal extracts against normally diluted standards (expected concentrations), leading to ~ 80% recovery of standards and thus, indicating that analyte detection may not be affected by 80% ethanol diluent and biological sample matrix diluted in the ratios of 1:25 and 1:50.

**Table S1.** List of studies on elephants* and other species** (using different sample matrices and assay kits) measuring different thyroid parameters (arranged based on the year of publication) related to different contexts.

| Serial no. | References | Species  (sexes, if specified) | Sample matrix | Context | Thyroid hormone parameters | Mean value | Minimum value | Maximum value | Assay kits |
| --- | --- | --- | --- | --- | --- | --- | --- | --- | --- |
| 1 | Brown et al., 2004* | Asian elephants (captive females) | Serum | Cycling | Total T4 (μg/dl) ± SE | 11.20 ± 0.57 | 8.62 | 14.54 | T3, T4 and TSH: Solid-phase and heterologous ^125^I radioimmunoassays (Coat-A-Count; Diagnostic Products Corporation, Los Angeles, CA) |
|  |  |  |  |  | Free T4 (ng/dl) | 1.01 ± 0.06 | 0.74 | 1.44 |  |
|  |  |  |  |  | Total T3 (ng/dl) | 123.95 ± 6.25 | 91.37 | 158.35 |  |
|  |  |  |  |  | Free T3 (pg/ml) | 1.93 ± 0.26 | 1.06 | 2.98 |  |
|  |  |  |  |  | TSH (ng/ml) | 0.75 ± 0.42 | 0.61 | 1.08 |  |
|  |  |  |  | Non cycling | Total T4 (μg/dl) | 11.12 ± 0.46 | 9.53 | 12.52 |  |
|  |  |  |  |  | Free T4 (ng/dl) | 0.87 ± 0.05 | 0.63 | 0.97 |  |
|  |  |  |  |  | Total T3 (ng/dl) | 126.72 ± 6.13 | 110.65 | 153.95 |  |
|  |  |  |  |  | Free T3 (pg/ml) | 1.39 ± 0.24 | 0.73 | 2.86 |  |
|  |  |  |  |  | TSH (ng/ml) | 0.97 ± 0.36 | 0.41 | 2.74 |  |
|  |  | African elephants (captive females) |  | Cycling | Total T4 (μg/dl) ± SE | 10.06 ± 0.36 | 7.58 | 12.25 |  |
|  |  |  |  |  | Free T4 (ng/dl) | 0.91 ± 0.03 | 0.72 | 1.1 |  |
|  |  |  |  |  | Total T3 (ng/dl) | 124.03 ± 4.30 | 99.34 | 148.07 |  |
|  |  |  |  |  | Free T3 (pg/ml) | 1.61 ± 0.27 | 0.7 | 3.49 |  |
|  |  |  |  |  | TSH (ng/ml) | 0.66 ± 0.15 | 0.42 | 1.27 |  |
|  |  |  |  | Non cycling | Total T4 (μg/dl) | 10.76 ± 0.41 | 8.45 | 16.56 |  |
|  |  |  |  |  | Free T4 (ng/dl) | 0.93 ± 0.04 | 0.72 | 1.46 |  |
|  |  |  |  |  | Total T3 (ng/dl) | 123.27 ± 4.38 | 89.49 | 177.49 |  |
|  |  |  |  |  | Free T3 (pg/ml) | 1.41 ± 0.21 | 0.41 | 3.68 |  |
|  |  |  |  |  | TSH (ng/ml) | 0.56 ± 0.14 | 0.37 | 0.86 |  |
| 2 | Brown et al., 2007 | Asian elephants (captive bulls) | Serum | Captive bulls | Total T4 (μg/dl) ± SE | 8.68 ± 0.65 |  |  | T3, T4 and TSH: Solid-phase and heterologous ^125^I radioimmunoassays (Coat-A-Count; Diagnostic Products Corporation, Los Angeles, CA) |
|  |  |  |  |  | Free T4 (ng/dl) | 0.72 ± 0.09 |  |  |  |
|  |  |  |  |  | Total T3 (ng/dl) | 112.15 ± 4.85 |  |  |  |
|  |  |  |  |  | Free T3 (pg/ml) | 1.72 ± 0.18 |  |  |  |
|  |  |  |  |  | TSH (ng/ml | 0.67 ± 0.10 |  |  |  |
|  |  | African elephants (captive bulls) |  |  | Total T4 (μg/dl) | 10.04 ± 0.68 |  |  |  |
|  |  |  |  |  | Free T4 (ng/dl) | 0.76 ± 0.06 |  |  |  |
|  |  |  |  |  | Total T3 (ng/dl) | 107.77 ± 5.23 |  |  |  |
|  |  |  |  |  | Free T3 (pg/ml) | 2.22 ± 0.38 |  |  |  |
|  |  |  |  |  | TSH (ng/ml) | 0.54 ± 0.05 |  |  |  |
| 3 | Thongtip et al., 2008* | Asian elephant (captive bulls) | Serum | 10–19 age group | T3 (ng/dl) ± SE | 224.86 ± 9.61 |  |  | Automated Chemiluminescence System (180, Bayer Corporation, Tarrytown, NY, USA) |
|  |  |  |  |  | T4 (μg/dl) | 11.51 ± 0.31 |  |  |  |
|  |  |  |  | 23–43 age group | T3 (ng/dl) | 169.54 ± 5.68 |  |  |  |
|  |  |  |  |  | T4 (μg/dl) | 9.81 ± 0.18 |  |  |  |
|  |  |  |  | 51–70 age group | T3 (ng/dl) | 138.95 ± 11.66 |  |  |  |
|  |  |  |  |  | T4 (μg/dl) | 10.63 ± 0.38 |  |  |  |
| 4 | Paudel et al., 2016* | Asian elephant (captive females) | Serum | TB-suspects | Free T3 (pg/ml) ± SD | 2.69 ± 0.34 | 1 | 4 | DiaMetra, Foligno, Italy; Free T3 (DKO037); Free T4 (DKO038) |
|  |  |  |  |  | Free T4 (ng/dl) | 0.69 ± 0.05 | 0.5 | 0.9 |  |
|  |  |  |  | Healthy | Free T3 (pg/ml) | 3.03 ± 0.25 | 1.6 | 3.9 |  |
|  |  |  |  |  | Free T4 (ng/dl) | 0.67 ± 0.04 | 0.4 | 0.8 |  |
| 5 | Chave et al., 2019 | Asian elephants (captive bulls) | Serum | Musth (group 1) | Total T4 (ug/dl) ± SD | 5.31 ± 1.41 | 2.63 | 8.77 | T3, T4, TSH: Solid-phase ^125^I radioimmunoassays (RIA) (Coat-A-Count; Siemens Healthcare Diagnostics Inc., Los Angeles, CA, USA) |
|  |  |  |  | Non-musth (group 1) |  | 7.03 ± 1.29 | 3.5 | 11.12 |  |
|  |  |  |  | Non-musth (group 2) |  | 6.65 ± 2.11 | 3.28 | 9.73 |  |
|  |  |  |  | Musth (group 1) | Free T4 (ng/dl) | 0.4 ± 0.15 | 0.16 | 0.88 |  |
|  |  |  |  | Non-musth (group 1) |  | 0.48 ± 0.18 | 0.21 | 0.96 |  |
|  |  |  |  | Non-musth (group 2) |  | 0.72 ± 0.16 | 0.51 | 1.12 |  |
|  |  |  |  | Musth (group 1) | Total T3 (ng/dl) | 82.02 ± 36.52 | 15.42 | 213.61 |  |
|  |  |  |  | Non-musth (group 1) |  | 76.81 ± 31.27 | 10.79 | 159.4 |  |
|  |  |  |  | Non-musth (group 2) |  | 126.51 ± 22.97 | 88.92 | 175.35 |  |
|  |  |  |  | Musth (group 1) | TSH (ng/ml) | 1.13 ± 0.66 | 0.16 | 3.53 |  |
|  |  |  |  | Non-musth (group 1) |  | 1.07 ± 0.53 | 0.1 | 3.42 |  |
|  |  |  |  | Non-musth (group 2) |  | 0.82 ± 0.35 | 0.1 | 1.45 |  |
| 6 | Szott et al., 2020 | African elephants (wild: males + females) | Faecal | Free-ranging female | T3 (μg/g) ± SD | 0.59 ± 0.27 | 0.23 | 1.83 | Arbor Assays (#K056) |
|  |  |  |  | Free-ranging male |  | 0.6 ± 0.32 |  |  |  |
|  |  |  |  | Identified individuals |  | 0.6 ± 0.27 | 0.26 | 1.37 |  |
|  |  |  |  | Body condition (2) |  | 0.18 |  |  |  |
|  |  |  |  | Body condition (3) |  | 0.3 |  |  |  |
| 7 | Keady et al., 2021 | Asian elephant (captive: males + females) | Serum | Comparative analyses | TSH (ng/ml) | 1.07 | 0.12 | 1.88 | T3, T4 and TSH: Solid-phase and heterologous ^125^I radioimmunoassays (Coat-A-Count; Diagnostic Products Corporation, Los Angeles, CA) |
|  |  |  |  |  | Total T3 (ng/dl) | 98.38 | 52.06 | 165.89 |  |
|  |  |  |  |  | Total T4 (μg/dl) | 10.2 | 6.48 | 12.64 |  |
|  |  |  |  |  | Free T4 (ng/dl) | 0.75 | 0.44 | 1.23 |  |
|  |  | African elephant (captive: males + females) |  |  | TSH (ng/ml) | 0.88 | 0.19 | 1.73 |  |
|  |  |  |  |  | Total T3 (ng/dl) | 85.52 | 38.04 | 117.64 |  |
|  |  |  |  |  | Total T4 (μg/dl) | 9.75 | 6.77 | 14.72 |  |
|  |  |  |  |  | Free T4 (ng/dl) | 0.83 | 0.49 | 1.17 |  |
| 8 | LaDue et al., 2022 | Asian elephants (captive + wild: bulls) | Faecal | Captive (low BCS) | T3 (ng/g) ± SE | 24.4 ± 1.2 |  |  | Arbor Assays (#K056) |
|  |  |  |  | Captive (high BCS) |  | 41.6 ± 0.9 |  |  |  |
|  |  |  |  | Wild (n = 37) | T3 (ng/g) | 7.74 | 3.9 | 14.78 |  |
|  |  |  |  | Captive (n = 840) |  | 35.32 | 3.31 | 118.56 |  |
| 9 | LaDue et al., 2023 | Asian elephants (captive bulls) | Faecal | Confirmed musth | T3 (ng/g) | 35.64 | 3.31 | 106.05 | Arbor Assays (#K056) |
|  |  |  |  | No-musth |  | 35.01 | 8.25 | 118.56 |  |
| 10 | Prado et al., 2023 | African elephants (captive females) | Serum | High prolactin | TSH (ng/ml) | 0.85 | 0.1 | 2.99 | T3, T4 and TSH: Solid-phase and heterologous ^125^I radioimmunoassays (Coat-A-Count; Diagnostic Products Corporation, Los Angeles, CA) |
|  |  |  |  |  | Total T4 (µg/dl) | 9.18 | 0.77 | 17.85 |  |
|  |  |  |  |  | Total T3 (ng/dl) | 77.5 | 21.73 | 130.02 |  |
|  |  |  |  |  | Free T4 (ng/dl) | 0.79 | 0.05 | 1.51 |  |
|  |  |  |  | Normal prolactin | TSH (ng/ml) | 0.97 | 0.1 | 2.53 |  |
|  |  |  |  |  | Total T4 (µg/dl) | 9.72 | 4.26 | 16.79 |  |
|  |  |  |  |  | Total T3 (ng/dl) | 87.77 | 18.16 | 171.02 |  |
|  |  |  |  |  | Free T4 (ng/dl) | 0.81 | 0.39 | 1.7 |  |
|  |  |  |  | Low prolactin | TSH (ng/ml) | 0.73 | 0.1 | 1.88 |  |
|  |  |  |  |  | Total T4 (µg/dl) | 10.07 | 6.07 | 18.36 |  |
|  |  |  |  |  | Total T3 (ng/dl) | 82.63 | 15.95 | 158.33 |  |
|  |  |  |  |  | Free T4 (ng/dl) | 0.82 | 0.4 | 1.29 |  |
| **Other animal species**:** | | | | | | | | | |
| 1 | Keech et al., 2010 | Steller sea lions | Faecal | TSH injections in four Steller sea lions | Baseline T3 (ng/g) ± SE | 583 ± 30 | 450 | 898 | ^125^I radioimmunoassay kit (Diagnostic Systems Laboratories, Inc., Webster, TX, catalog #DSL-3100) |
|  |  |  |  |  | Baseline T4 (ng/g) | 2012 ± 134 | 1489 | 4276 |  |
|  |  |  |  |  | Peak T3 (ng/g) | 752 ± 54.24 |  |  |  |
|  |  |  |  |  | Peak T4 (ng/g) | 2842 ± 492.18 |  |  |  |
|  |  |  |  | Older females | Baseline T3 (ng/g) | 514 ± 14 |  |  |  |
|  |  |  |  | Younger females |  | 652 ± 43 |  |  |  |
| 2 | Gobush et al., 2014 | Wild Hawaiian monk seals | Faecal | Overall values | T3 (ng/g) | 412.9 ± 47.0 | 29.7 | 3313.4 | ^125^I radioimmunoassay kit (Diagnostic Systems Laboratories, Inc., Webster, TX, catalog #DSL-3100) |
|  |  |  |  | Gained weight (8kg) |  | 413.3 |  |  |  |
|  |  |  |  | Gained weight (7kg) |  | 302.7 |  |  |  |
|  |  |  |  | Gained weight (3kg) |  | 204 |  |  |  |
|  |  |  |  | Molting |  | ~1100-1200 |  |  |  |
|  |  |  |  | Pre-molting |  | 200-300 |  |  |  |
|  |  |  |  | No molting |  | 200-300 |  |  |  |
|  |  |  |  | Adult |  | ~350 |  |  |  |
|  |  |  |  | Immature |  | ~300 |  |  |  |
| 3 | Cristóbal-Azkarate et al., 2016 | Wild Barbary macaques | Faecal | Provisioned group | T3 (ng/g) | 253.21 |  |  | T3 enzyme-immunoassay from IBL International (RE55251) |
|  |  |  |  | Wild feeding group |  | 225.96 |  |  |  |
| 4 | Gesquiere et al., 2018 | Wild baboon | Faecal | Wild baboons | T3 (ng/g) ± SD | 141 ± 56 | 28 | 349 | ^125^I Total T3 radioimmunoassay (RIA) (#06B254216, MP Biomedicals, Costa Mesa, CA) |
|  |  |  |  | Captive baboons |  | 454 ± 143 | 276 | 704 |  |
| 5 | Hunninck et al., 2020 | Wild impala | Faecal | Highest NDVI values | T3 (ng/g) (CI) | 566 | 456 | 703 | ^125^I Total T3 radioimmunoassay (RIA) (#06B254216, MP Biomedicals, Costa Mesa, CA) |
|  |  |  |  | Lowest NDVI values |  | 1018 | 876 | 1183 |  |
|  |  |  |  | Highest temperature |  | 414 | 338 | 507 |  |
|  |  |  |  | Lowest temperature |  | 1358 | 1147 | 1609 |  |
|  |  |  |  | Higher disturbance |  | 691 | 599 | 797 |  |
|  |  |  |  | Lesser disturbance |  | 869 | 786 | 960 |  |
|  |  |  |  | Highest fGCM |  | 935 | 772 | 1133 |  |
|  |  |  |  | Lowest fGCM |  | 674 | 562 | 808 |  |
| 6 | Lemos et al., 2020 | North Pacific Gray whales | Faecal | Overall | T3 (ng/g) ± SD | 142.41 ± 320.28 | 1.28 | 2134.86 | Arbor Assays (#K056) |
|  |  |  |  | Immature male |  | 49.58 ± 37.86 | 8.44 | 100.02 |  |
|  |  |  |  | Mature male |  | 192.57 ± 461.10 | 1.28 | 2134.86 |  |
|  |  |  |  | Immature female |  | 48.42 ± 17.56 | 28.14 | 58.82 |  |
|  |  |  |  | Resting female |  | 8 ± 152.97 | 3.99 | 624.5 |  |
|  |  |  |  | Pregnant female |  | 18.57 ± 9.64 | 10.77 | 29.35 |  |
|  |  |  |  | Lactating female |  | 65.88 |  |  |  |
| 7 | Mondol et al., 2020 | Captive tigers | Faecal | TSH challenge pre-basal (female) | T3 (ng/g) | ~400 |  |  | ^125^I Total T3 radioimmunoassay (RIA) (#06B254216, MP Biomedicals, Costa Mesa, CA) |
|  |  |  |  | TSH challenge pre-basal (male) |  | 200-400 |  |  |  |
|  |  |  |  | TSH challenge peak (female) |  | ~1200 |  |  |  |
|  |  |  |  | TSH challenge peak (male) |  | 800-1000 |  |  |  |
|  |  |  |  | TSH challenge post-basal (female) |  | 200-300 |  |  |  |
|  |  |  |  | TSH challenge post-basal (male) |  | 200-400 |  |  |  |
| 8 | Patel et al., 2021 | Wild tigers | Faecal | Rajaji tiger reserve | T3 (ng/g) per organic dry matter ± SD | 8.26 ± 0.99 |  |  | Arbor Assays (#K056) |
|  |  |  |  | Corbett tiger reserve |  | 8.67 ± 0.77 |  |  |  |
|  |  |  |  | Dudhwa and Pilibhit tiger reserve |  | 7.92 ± 0.89 |  |  |  |
|  |  |  |  | Valmiki tiger reserve |  | 8.62 ± 1.23 |  |  |  |
|  |  | Captive Asiatic lions |  | Pre-corrective method |  | 7.82 ± 0.29 |  |  |  |
|  |  |  |  | Post-corrective method |  | 7.89 ± 0.30 |  |  |  |

*References related to elephants which are not in the main text:

1. Brown, J.L., Walker, S.L. and Moeller, T., 2004. Comparative endocrinology of cycling and non-cycling Asian (*Elephas maximus*) and African (*Loxodonta africana*) elephants. *General and comparative endocrinology*, 136(3), pp.360-370.
2. Thongtip, N., Saikhun, J., Mahasawangkul, S., Kornkaewrat, K., Pongsopavijitr, P., Songsasen, N. and Pinyopummin, A., 2008. Potential factors affecting semen quality in the Asian elephant (*Elephas maximus*). *Reproductive Biology and Endocrinology*, 6, pp.1-9.
3. Paudel, S., Brown, J.L., Thapaliya, S., Dhakal, I.P., Mikota, S.K., Gairhe, K.P., Shimozuru, M. and Tsubota, T., 2016. Comparison of cortisol and thyroid hormones between tuberculosis-suspect and healthy elephants of Nepal. *Journal of Veterinary Medical Science*, 78(11), pp.1713-1716.

**References related to other animals:

1. Keech, A.L., Rosen, D.A.S., Booth, R.K., Trites, A.W. and Wasser, S.K., 2010. Fecal triiodothyronine and thyroxine concentrations change in response to thyroid stimulation in Steller sea lions (Eumetopias jubatus). *General and Comparative Endocrinology*, *166*(1), pp.180-185.
2. Gobush, K.S., Booth, R.K. and Wasser, S.K., 2014. Validation and application of noninvasive glucocorticoid and thyroid hormone measures in free-ranging Hawaiian monk seals. *General and Comparative Endocrinology*, 195, pp.174-182.
3. Cristóbal-Azkarate, J., Maréchal, L., Semple, S., Majolo, B. and MacLarnon, A., 2016. Metabolic strategies in wild male Barbary macaques: evidence from faecal measurement of thyroid hormone. *Biology Letters*, 12(4), p.20160168.
4. Gesquiere, L.R., Pugh, M., Alberts, S.C. and Markham, A.C., 2018. Estimation of energetic condition in wild baboons using fecal thyroid hormone determination. *General and comparative endocrinology*, 260, pp.9-17.
5. Hunninck, L., Jackson, C.R., May, R., Røskaft, E., Palme, R. and Sheriff, M.J., 2020. Triiodothyronine (T3) levels fluctuate in response to ambient temperature rather than nutritional status in a wild tropical ungulate. *Conservation Physiology*, 8(1), p.coaa105.
6. Lemos, L.S., Olsen, A., Smith, A., Chandler, T.E., Larson, S., Hunt, K. and Torres, L.G., 2020. Assessment of fecal steroid and thyroid hormone metabolites in eastern North Pacific gray whales. *Conservation Physiology*, 8(1), p.coaa110.
7. Mondol, S., Booth, R.K. and Wasser, S.K., 2020. Fecal stress, nutrition and reproductive hormones for monitoring environmental impacts on tigers (*Panthera tigris*). *Conservation physiology*, 8(1), p.coz091.
8. Patel, S.K., Biswas, S., Goswami, S., Bhatt, S., Pandav, B. and Mondol, S., 2021. Effects of faecal inorganic content variability on quantifying glucocorticoid and thyroid hormone metabolites in large felines: implications for physiological assessments in free-ranging animals. *General and Comparative Endocrinology*, 310, p.113833.

**Table S2.** Pairwise contrasts of estimated marginal means (EMMs) across population, sex, and age categories for fGCM and fT3 levels in free-ranging Asian elephants, using the ‘*emmeans*’ function from the ‘emmeans’ package in R*, at a 95% confidence level, where *P*-value was adjusted using the Tukey method for multiple comparisons.

| Predictors | Hormone types | Contrast | Estimate | SE | df | t ratio | p-value |
| --- | --- | --- | --- | --- | --- | --- | --- |
| Population | fGCM | (Central) - (North-eastern 1) | **0.35** | **0.14** | **146** | **2.53** | **0.04** |
|  |  | (Central) - (North-eastern 2) | **0.81** | **0.12** | **146** | **6.54** | **<0.0001** |
|  |  | (Central) - (Southern) | **0.41** | **0.17** | **146** | **2.40** | **0.02** |
|  |  | (North-eastern 1) - (North-eastern 2) | **0.46** | **0.14** | **146** | **3.43** | **0.004** |
|  |  | (North-eastern 1) - (Southern) | 0.06 | 0.18 | 146 | 0.34 | 1.00 |
|  |  | (North-eastern 2) - (Southern) | -0.41 | 0.17 | 146 | -2.36 | 0.10 |
|  | fT3 | (Central) - (North-eastern 1) | **-0.44** | **0.10** | **100** | **-5.20** | **<0.0001** |
|  |  | (Central) - (North-eastern 2) | **-0.50** | **0.10** | **100** | **-5.72** | **<0.0001** |
|  |  | (North-eastern 1) - (North-eastern 2) | -0.05 | 0.10 | 100 | -0.57 | 0.84 |
| Sex | fT3 | (Females) - (Males) | 0.12 | 0.10 | 100 | 1.69 | 0.25 |
|  |  | (Females) - (Unidentified) | -0.23 | 0.22 | 100 | -1.10 | 0.55 |
|  |  | (Males) - (Unidentified) | -0.35 | 0.22 | 100 | -1.62 | 0.24 |
| Age | fT3 | (Adults) - (Subadults) | **0.31** | **0.10** | **100** | **3.50** | **0.001** |

******formula: emmeans (model, pairwise ~ Predictors)*

**Table S3.** Total forest cover area, forest types, and landscape metrics for the four study landscapes, categorized by district boundary (DB) and forest division boundary including a 5 km buffer area (FB5). The highest values are highlighted in red, while the lowest values are highlighted in blue.

| Landscape metrics | Study landscape | | | |
| --- | --- | --- | --- | --- |
|  | North-eastern-1 | North-eastern-2 | Central | Southern |
| Forest cover area (sq km): |  |  |  |  |
| (a) across DB | 1331.9 | 1577.6 | 2200.4 | 1526.8 |
| (b) across FB5 | 1038.2 | 1520.6 | 1694.1 | 1001.3 |
|  |  |  |  |  |
| Forest types (sq km) (across DB): |  |  |  |  |
| Scrub (<10% canopy density) | 19.7 | 15.9 | 21.0 | 70.5 |
| Open Forest (10% to 40% canopy density) | 905.7 | 807.1 | 1337.8 | 567.3 |
| Moderately Dense Forest (40% to 70% canopy density) | 115.1 | 326.0 | 601.1 | 740.7 |
| Very Dense Forest (>70% canopy density) | 294.9 | 429.2 | 242.9 | 152.8 |
| Total forest cover | 1335.5 | 1578.2 | 2202.8 | 1531.3 |
|  |  |  |  |  |
| Landscape metrics: |  |  |  |  |
| Edge length (km): (a) across DB | 6697.2 | 7854.5 | 19116.4 | 10983.4 |
| (b) across FB5 | 3806.4 | 6682.9 | 10700.5 | 5729.0 |
| Edge density (meters/ hectares): (a) across DB | 19.5 | 27.6 | 20.1 | 16.0 |
| (b) across FB5 | 18.8 | 26.3 | 19.1 | 28.0 |
| Patch number: (a) (across DB) | 2454.0 | 2694.0 | 7395.0 | 2986.0 |
| (b) (across FB5) | 1141.0 | 1919.0 | 3066.0 | 674.0 |
| Patch density (number/100 hectares): (a) across DB | 0.7 | 0.9 | 0.8 | 0.4 |
| (b) across FB5 | 0.6 | 0.8 | 0.5 | 0.3 |
| Largest patch index (%): (a) across DB | 7.7 | 25.3 | 2.4 | 8.3 |
| (b) across FB5 | 11.8 | 28.3 | 4.0 | 27.7 |
| Core area (%): (a) across DB | 28.1 | 40.4 | 12.3 | 12.9 |
| (b) across FB5 | 40.4 | 44.9 | 19.1 | 31.0 |

**Table S4.** Media reports and publicly uploaded videos showing the ‘*hula party*’* practice in the central landscapes (southern West Bengal).

| SI No. | Coverage date | Year | Media coverage: Title | Links |
| --- | --- | --- | --- | --- |
| 1 | 22nd Dec 2016 | 2016 | Hindustan Times: Elephant chasers feel cash crunch heat, more lives at risk | https://www.hindustantimes.com/kolkata/elephant-chasers-feel-cash-crunch-heat-more-lives-at-risk/story-ZFyRrbDcv89jAzr3fVwiaK.html |
| 2 | 1st Jan 2018 | 2018 | Mint: Living with the elephants | [Living with the elephants \| Mint Lounge (livemint.com)](https://lifestyle.livemint.com/news/talking-point/living-with-the-elephants-111646917110902.html) |
| 3 | 30th July 2018 | 2018 | Times of India: Conservationists move SC against use of hulla parties | https://timesofindia.indiatimes.com/city/kolkata/conservationists-move-sc-against-use-of-hulla-parties/articleshow/65204690.cms |
| 4 | 28th July 2019 | 2019 | YouTube: Living with Elephants | https://www.youtube.com/watch?v=2WRMXrGa5_g&t=870s&ab_channel=SashidharVempala |
| 5 | 15th April 2021 | 2021 | The Week: Tusker kills man in West Bengal's Jhargram | https://www.theweek.in/wire-updates/national/2021/04/15/ces8-wb-tusker.html |
| 6 | 2nd August 2022 | 2022 | Youtube: Hula Party driving away Elephants at Nayagram 2 Jhargram | https://www.youtube.com/watch?v=_z_95pKbbFY&ab_channel=KanchanPathak |
| 7 | 12th Jan 2022 | 2022 | YouTube: The Hula party is chasing _ Elephants_ with fire | https://www.youtube.com/watch?v=PHeB4Y2m5Lw&ab_channel=REEFLIXNEWS |
| 8 | 14th June 2022 | 2022 | YouTube: At night, the people of the forest are chasing the wild elephants with the hula party team | https://www.youtube.com/watch?v=rglC8qoCYoY&ab_channel=WBELEPHANT |
| 9 | 16th Oct 2022 | 2022 | YouTube: Elephant charge the Man Hula party jump into the river after being chased by wild elephants | https://www.youtube.com/watch?v=goool_IqRJM&ab_channel=BTELECOM |
| 10 | 22nd Jan 2022 | 2022 | YouTube: wild life Elephant 🐘 🐘 Bankura West Bengal Elephant 🐘 ♥ Kids Come Near To Village Area. | https://www.youtube.com/shorts/l_NyM_apBZ8 |
| 11 | 20th April 2023 | 2023 | The Hindu: Video of hula party violently throwing flaming torches at elephant surfaces in south Bengal | [Video of hula party violently throwing flaming torches at elephant surfaces in south Bengal - The Hindu](https://www.thehindu.com/news/national/other-states/video-of-hula-party-violently-throwing-flaming-torches-at-elephant-surfaces-in-south-bengal/article66756565.ece) |
| 12 | 29th July 2023 | 2023 | Times of India: Villagers on alert as hula parties go on strike in Jangalmahal | [Villagers on alert as hula parties go on strike in Jangalmahal \| Kolkata News - Times of India (indiatimes.com)](https://timesofindia.indiatimes.com/city/kolkata/villagers-on-alert-as-hula-parties-go-on-strike-in-jangalmahal/articleshow/102219266.cms) |
| 13 | 18th April 2023 | 2023 | YouTube: Hula Party and elephants | https://www.youtube.com/shorts/Amyy8R0pumM |
| 14 | 6th Jan 2024 | 2024 | The Telegraph: 22-year-old hula party member trampled to death by wild elephant in Jhargram’s Chitalbani village | https://www.telegraphindia.com/west-bengal/22-year-old-hula-party-member-trampled-to-death-by-wild-elephant-in-jhargrams-chitalbani-village/cid/1991856 |
|  |  | 2024 | The Telegraph: Youth diverting elephant herd killed | https://www.telegraphindia.com/west-bengal/youth-diverting-elephant-herd-killed/cid/1929327 |
| 15 | 20^th^ August 2024 | 2024 | Uproar after elephant attacked with a flaming spear dies in West Bengal forest | https://www.hindustantimes.com/india-news/uproar-after-elephant-attacked-with-a-flaming-spear-dies-in-west-bengal-forest-101724093324177.html |
| 16 | 27^th^ October 2024 | 2024 | Torches and tension on elephant corridors | https://www.thehindu.com/news/national/west-bengal/torches-and-tension-on-elephant-corridors/article68797119.ece |

**The ‘Hula party’ is an operation carried out by local villagers in the southern West Bengal to chase away elephants. During this operation, 20-25 villagers gather and use fireballs, firecrackers and “hula” sticks (wooden sticks with a gunny rag doused in oil or mobil, tied to one end and set aflame, often with a sharp metal spearhead sticking out in some cases) to deter and scare elephants away. Media information was collected and compiled through a systematic search using the keywords "hula party," "elephant driving operations in India," "elephant-human conflict in West Bengal," "elephant-human conflict in India," and "hula party in Bengal, India" on the Google search engine.*

**Table S5.** Elephant age-class wise distribution of sample size across population and sex categories for fGCM and fT3 analyses.

| Sample distribution | Faecal GC metabolites (n = 153) | | Faecal T3 (n = 107) | |
| --- | --- | --- | --- | --- |
|  | Adult | Subadult | Adult | Subadult |
| Population-wise: | | | | |
| North-eastern-1 | 30 | 5 | 29 | 4 |
| North-eastern-2 | 40 | 5 | 32 | 4 |
| Central | 35 | 17 | 29 | 9 |
| Southern* | 21 | 0 | 0 | 0 |
| Sex-wise: | | | | |
| Female | 70 | 13 | 53 | 13 |
| Male | 39 | 4 | 34 | 4 |
| Unidentified | 17 | 10 | 3 | 0 |

**Reference samples from Pokharel et al., 2019*

**Table S6.** Model selection table representing top five GLMs generated from the global model*, to understand the influence of potential predictors and their interactions on fGCM levels in free-ranging Asian elephants, using the function ‘dredge’ in the package ‘MuMIn’ and ranked based on their AIC parameters. The top model (having lowest AIC, Delta score and highest AIC weight) has been marked bold.

| Model # | Intercept | Predictors | | | | | | | | df | logLik | AIC | Delta | Weight |
| --- | --- | --- | --- | --- | --- | --- | --- | --- | --- | --- | --- | --- | --- | --- |
|  |  | Age | C/N ratio | Population (Pop) | Sex | Age*Pop | Age*Sex | Pop*Sex | Pop*Age*Sex |  |  |  |  |  |
| **7** | **-0.92** |  | **0.02** | **+** |  |  |  |  |  | **6** | **-12.0** | **36.1** | **0.0** | **0.5** |
| 8 | -0.91 | + | 0.02 | + |  |  |  |  |  | 7 | -12.0 | 38.3 | 2.2 | 0.2 |
| 15 | -0.91 |  | 0.02 | + | + |  |  |  |  | 8 | -11.0 | 39.0 | 2.5 | 0.2 |
| 16 | -0.91 | + | 0.02 | + | + |  |  |  |  | 9 | -11.0 | 41.0 | 5.0 | 0.1 |
| 24 | -0.90 | + | 0.02 | + |  | + |  |  |  | 9 | -11.1 | 42.0 | 5.4 | 0.03 |

**global model = glm (formula = fGCM ~ Age + Sex + C/N ratio + Population + Age x Sex x Population, family = Gamma (link = log), data)*

**Table S7.** Model selection table representing top five GLMs generated from the global model*, to understand the influence of potential predictors and their interactions on fT3 levels in free-ranging Asian elephants, using the function ‘dredge’ in the package ‘MuMIn’ and ranked based on their AIC parameters. The top model (having lowest AIC, Delta score and highest AIC weight) has been marked bold.

| Model # | Intercept | Predictors | | | | | | | | df | logLik | AIC | Delta | Weight |
| --- | --- | --- | --- | --- | --- | --- | --- | --- | --- | --- | --- | --- | --- | --- |
|  |  | Age | C/N ratio | Population (Pop) | Sex | Age*Pop | Age*Sex | Pop*Sex | Pop*Age*Sex |  |  |  |  |  |
| **16** | **-1.60** | **+** | **0.01** | **+** | **+** |  |  |  |  | **8** | **86.0** | **-155.0** | **0.0** | **0.3** |
| 8 | -1.60 | + | 0.02 | + |  | + |  | + |  | 6 | 83.3 | -154.0 | 0.7 | 0.2 |
| 32 | -1.60 | + | 0.02 | + | + | + |  | + |  | 10 | 88.0 | -153.0 | 2.0 | 0.1 |
| 48 | -1.61 | + | 0.02 | + | + | + |  |  |  | 9 | 86.2 | -152.4 | 2.1 | 0.1 |
| 80 | -1.54 | + | 0.02 | + | + | + | + |  |  | 10 | 87.0 | -152.0 | 3.0 | 0.1 |

**global model = glm (formula = fT3 ~ Age + Sex + C/N ratio + Population + Age x Sex x Population, family = Gamma (link = log), data)*
